# Using Community Composition and Successional Theory to Guide Site‐Specific Coral Reef Management

**DOI:** 10.1111/gcb.70050

**Published:** 2025-01-28

**Authors:** Orion S. McCarthy, Emily L. A. Kelly, Anela K. Akiona, Samantha M. Clements, Tatiana Martinez, Nicole E. Pedersen, Cole Peralto, Sarah L. Romero, Mitchell H. Smelser, Kristy Wong Stone, Russell T. Sparks, Jennifer E. Smith

**Affiliations:** ^1^ Scripps Institution of Oceanography UC San Diego La Jolla California USA; ^2^ World Economic Forum San Francisco California USA; ^3^ Hawai'i Division of Aquatic Resources Maui Hawaii USA

**Keywords:** benthic communities, climate change, community ecology, coral reef, Hawaii, landscape ecology, photogrammetry, succession

## Abstract

High spatial or temporal variability in community composition makes it challenging for natural resource managers to predict ecosystem trajectories at scales relevant to management. This is commonly the case in nearshore marine environments, where the frequency and intensity of disturbance events vary at the sub‐kilometer to meter scale, creating a patchwork of successional stages within a single ecosystem. The successional stage of a community impacts its stability, recovery potential, and trajectory over time in predictable ways. Here we demonstrate the value of successional theory for interpreting fine‐scale community heterogeneity using Hawaiian coral reefs as a case study. We tracked benthic community dynamics on 36 forereefs over a 6‐year period (2017–2023) that captures impacts from high surf events, a marine heatwave, and unprecedented shifts in human behavior due to the COVID‐19 pandemic. We document high spatial variation in benthic community composition that was only partially explained by island and environmental regime. Through hierarchical clustering, we identify three distinct community types that appear to represent different successional stages of reef development. Reefs belonging to the same community type exhibited similar rates of change in coral cover and structural complexity over time, more so than reefs located on the same island. Importantly, communities that were indicative of early succession (low coral cover reefs dominated by stress‐tolerant corals) were most likely to experience an increase in coral cover over time, while later‐stage successional communities were more likely to experience coral decline. Our findings highlight the influence of life history and successional stage on community trajectories. Accounting for these factors, not simply overall coral cover, is essential for designing effective management interventions. Site‐specific management that accounts for a community's unique composition and history of disturbance is needed to effectively conserve these important ecosystems.

## Graphical abstract

Coral reef benthic communities are highly variable over small distances, even when accounting for environmental conditions. We used a time series of coral reef 3D models to track reef change over time in Maui Nui, Hawai'i. We identified three distinct reef communities based on similarities in 3D habitat structure and the identity and arrangement of benthic taxa. These reef types changed in similar ways over time, more so than nearby reefs facing similar environmental conditions, and may represent distinct successional stages. Following the Biological Legacies Model of succession, resource managers should leverage community composition to inform site‐specific management for coral reefs.

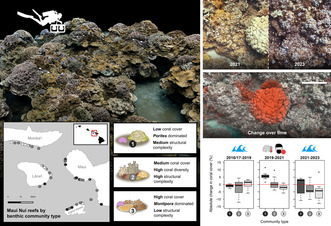

## 1 Introduction

The question of how biological communities vary in space and time, and what mechanisms are responsible for such variation, is increasingly relevant for natural resource managers, who are tasked with maintaining ecosystem stability in an era of heightened anthropogenic impact. Management is more effective when resource managers understand the primary drivers of community variation within their jurisdiction (Pullin and Knight 2003; Wedding et al. 2018). Once locally relevant drivers of community variation have been identified, managers can design targeted conservation interventions that reduce ecosystem degradation and promote recovery (Anthony et al. 2015; Donovan et al. 2023; Gove et al. 2023; Sugihara et al. 2012).

To integrate a community ecology perspective in conservation decision‐making, resource managers often rely on data from long‐term monitoring programs. These observational data provide important context about community variation, stability, and trajectory over time (Hughes and Connell 1999; Lindenmayer and Likens 2009; Moritz et al. 2021; Rodgers et al. 2021). However, applying monitoring data to identify causal drivers of community variation can be deceptively challenging, due in part to uncertainties related to scale (Hatcher, Imberger, and Smith 1987; Levin 1992; Menge and Olson 1990; Murdoch and Aronson 1999). How far in space and time can observations about ecosystem condition be extrapolated? Do neighboring sites represent replicates of the same community or different communities? Do observed patterns represent baseline conditions, recovery from disturbance, or transient dynamics (Hock et al. 2024)?

The answers to these questions depend in part on the spatial and temporal scales of the monitoring program and the ecosystem under study. Depending on the scale of observation, community dynamics may appear driven by environmental factors or appear decoupled, instead reflecting biotic feedbacks that manifest as regular pattern formation and spatial autocorrelation (Ford et al. 2021; Legendre, Gauthier, and Centre‐ville 2014; Rietkerk and van de Koppel 2008). Both of these observations can be true simultaneously, since ecological systems are structured by both biotic and abiotic processes that operate at different spatial and temporal scales (Hatcher, Imberger, and Smith 1987; Holling 1992; Kotliar and Wiens 1990; Krummel et al. 1987; Levin 1992). For example, the distribution of organisms in a habitat may be driven simultaneously by competition over the scale of meters and by climatic variation over the scale of hundreds of kilometers (Barott et al. 2012; Best et al. 2007; Meier et al. 2011; Ritchie et al. 2009).

Similarly, community recovery following a disturbance will depend on the scale of the disturbance in both magnitude and duration, but also the heterogeneity of disturbance impacts and the spatial distribution and composition of the surviving community (Connell 1997; Dietzel et al. 2021; Franklin et al. 2000; Haire and McGarigal 2009; Turner et al. 1998). This is especially true for benthic communities on coral reefs, which exhibit high spatial and temporal variation due to their location in dynamic nearshore environments (Friedlander et al. 2003; Gouezo et al. 2019; Hughes and Connell 1999).

Succession on coral reefs is frequently reset by disturbance events, which foster diversity in both space and time (Gouezo et al. 2019; Grigg 1983; Jouval et al. 2020). According to the intermediate disturbance hypothesis, frequent and moderate‐strength disturbance events prevent competitive exclusion and allow diverse communities of organisms to coexist (Connell 1978; Dollar 1982; Grigg 1983; Mouillot et al. 2013; Rogers 1993). In addition to promoting species diversity, patchy disturbances can also lead to high spatial heterogeneity within the same reef tract. For example, exposed coastlines will bear the brunt of wave damage from incoming storms (Dietzel et al. 2021), as will shallower sites (Dollar 1982), compared to more sheltered locations. Sediment deposition onto corals may be concentrated at stream outflows (Jokiel et al. 2014; Minton et al. 2022) or on reefs with higher densities of macroalgae (Stamski and Field 2006). Water flow and turbidity can moderate coral bleaching and mortality during marine heatwaves at the scale of 100s of meters to kilometers (Carlson et al. 2022; Morais et al. 2023; Yadav et al. 2023). Furthermore, the life history strategies of different taxa can create meter‐scale variation in disturbance impacts (Burns et al. 2016; Jouval et al. 2020; Lu et al. 2020; Maragos and Grigg 1974), producing a complex patchwork of successional stages with different characteristics (Gouezo et al. 2019; Hughes and Connell 1999). Beyond coral reefs, this pattern was famously documented in forest ecosystems following the eruption of Mount St. Helens (Franklin et al. 2000) and has been termed the Biological Legacies Model of succession (Pulsford, Lindenmayer, and Driscoll 2016). Following this model, the dominant life history traits and community composition before disturbance can predict the development and trajectory of the post‐disturbance community.

Following the Biological Legacies Model, an understanding of a community's successional stage can provide useful context for natural resource managers (Franklin et al. 2000; Gouezo et al. 2021; Imai et al. 2014). Different successional stages on coral reefs essentially represent different community types, or reef regimes, each characterized by its own cast of species and rates of competition, recruitment, habitat structural complexity, and herbivory (Connell 1997; Dudgeon et al. 2010; Rogers 1993). Temporal trends and recovery potential will also differ among reef regimes (Cresswell et al. 2023; Crisp, Tebbett, and Bellwood 2022; Donovan et al. 2018; Dudgeon et al. 2010). For example, coral growth in recently disturbed low‐density communities will be controlled primarily by environmental conditions, whereas interspecific competition will play a larger role in later‐stage successional communities with higher coral densities (Mouillot et al. 2013; Rogers 1993). Thus, different reef regimes will likely have characteristic responses to acute and chronic anthropogenic impacts as well as conservation interventions (Jouffray et al. 2019).

Studies that identify and map different coral reef regimes have mostly spanned large archipelagic scales (Donovan et al. 2018; Jouffray et al. 2015, 2019; Smith et al. 2016). An archipelago perspective is particularly useful for identifying regimes that are structured by strong environmental gradients, such as variation between leeward and windward coastlines (Donovan et al. 2018; Storlazzi et al. 2005; Williams et al. 2013). Archipelagic‐scale studies are also useful for identifying locations where human impacts have decoupled expected bio‐physical relationships (Sandin et al. 2008; Smith et al. 2016; Williams et al. 2015). However, fewer studies have sought to identify distinct reef regimes across finer spatial gradients with limited oceanographic variability (but see DeVantier et al. 1998), even though such intra‐island studies are more consistent with the jurisdictional purview of most natural resource management agencies (Sandin et al. 2022). Spatial variation in community composition can be substantial among reefs on the same island (kilometer scale; Ford et al. 2021; Friedlander et al. 2003; Murdoch and Aronson 1999; Sandin et al. 2022) or even within the same reef (meter scale; Edwards et al. 2017; Hughes et al. 1999; Ross et al. 2012). Heterogeneity of reef regimes at these scales could indicate the need for a site‐specific approach to management.

Failing to account for sources of community variation at scales relevant to management can reduce management efficacy (Kotliar and Wiens 1990; Pullin and Knight 2003; Rogers et al. 2015). However, it can be difficult to design an appropriate monitoring program without prior knowledge of the processes driving community variation and the scales over which those processes operate (Gouezo et al. 2021; Hatcher, Imberger, and Smith 1987; Wiens 1989). Conservation technology that allows resource managers to study community structure at multiple scales simultaneously can help. One such technology is large‐area imagery, also known as Structure from Motion photogrammetry. Large‐area imagery surveys can reconstruct 3D models of benthic environments covering 100 s of square meters with millimeter‐scale resolution (Edwards et al. 2023). When these data are collected repeatedly at fixed sites, the added temporal dimension creates new opportunities for multi‐scale studies of community variation in space and time (McCarthy et al. 2023).

Here, we utilize a timeseries of 3D coral reef models from the Maui Nui region of the Hawaiian Islands to (1) describe spatial and temporal variation in benthic community composition; (2) determine if reefs with similar community composition display similar successional trajectories; and (3) identify the drivers of coral cover and structural complexity change. The spatial scale of this study corresponds to the management jurisdiction of the Maui office of the Hawai'i Division of Aquatic Resources and complements statewide long‐term monitoring conducted via the Hawai'i Coral Reef Assessment and Monitoring Program (CRAMP; Brown et al. 2008; Rodgers et al. 2015; Sparks et al. 2015).

## 2 Methods

### 2.1 Study Design

The Hawaiian islands of Maui, Kaho'olawe, Lāna'i, and Moloka'i were connected as recently as 21,000–12,000 years ago, forming a single island called Maui Nui (Field et al. 2019; Grigg et al. 2002; Price and Elliott‐Fisk 2004). Today, these neighboring islands are separated by a series of shallow (< 300 m) channels that are largely protected from open ocean swell (Storlazzi et al. 2005). Due in part to reduced wave exposure, reef tracts in the region have higher coral cover than elsewhere in the Hawaiian Islands (Field et al. 2019). However, each island is also exposed to a unique combination of anthropogenic impacts. Maui is home to > 140,000 residents and hosts > 2 million tourists per year (Hawaii Tourism Authority 2023), and as a result, Maui's reefs experience more nutrient pollution, fishing, and other chronic stressors compared to the neighboring islands (Brown 2004; Dailer et al. 2010; Maynard et al. 2019; Smith, Runcie, and Smith 2005; Vermeij et al. 2010; Weng et al. 2023). Moloka'i and Lāna'i host smaller communities (< 10,000 people), while Kaho'olawe is uninhabited with restricted access, including for fishing, due to the presence of unexploded ordinances (Jokiel, Cox, and Crosby 1995). Although these islands have a smaller human population than Maui, their nearshore environments experience chronic sedimentation due to hillside erosion and overgrazing by invasive ungulates (Jokiel, Cox, and Crosby 1995; Jokiel et al. 2014; Minton et al. 2022; Stamski and Field 2006; Takesue 2010), and in the case of Kaho'olawe, severe degradation from decades of munitions testing by the US military (Jokiel, Cox, and Crosby 1995).

We surveyed 36 fixed long‐term monitoring sites in the Maui Nui region (Figure S1). Benthic data from these surveys, as well as videos of select 3D models, are available online in an interactive map and on Dryad (McCarthy et al. 2025). Surveys were completed as part of the 100 Island Challenge, a broader effort to document global patterns of coral reef condition and change over time (Naughton et al. 2015; Sandin et al. 2020). Sites were confined to forereef habitat with leeward exposure at approximately 10 m depth to decrease environmental differences and enable fine‐scale comparisons between sites, with eight to twelve sites selected per island. Several sites on Maui and Moloka'i are positioned close to or overlap with fixed long‐term CRAMP transects maintained by the state of Hawai'i (Figure S2).

**FIGURE 1:**
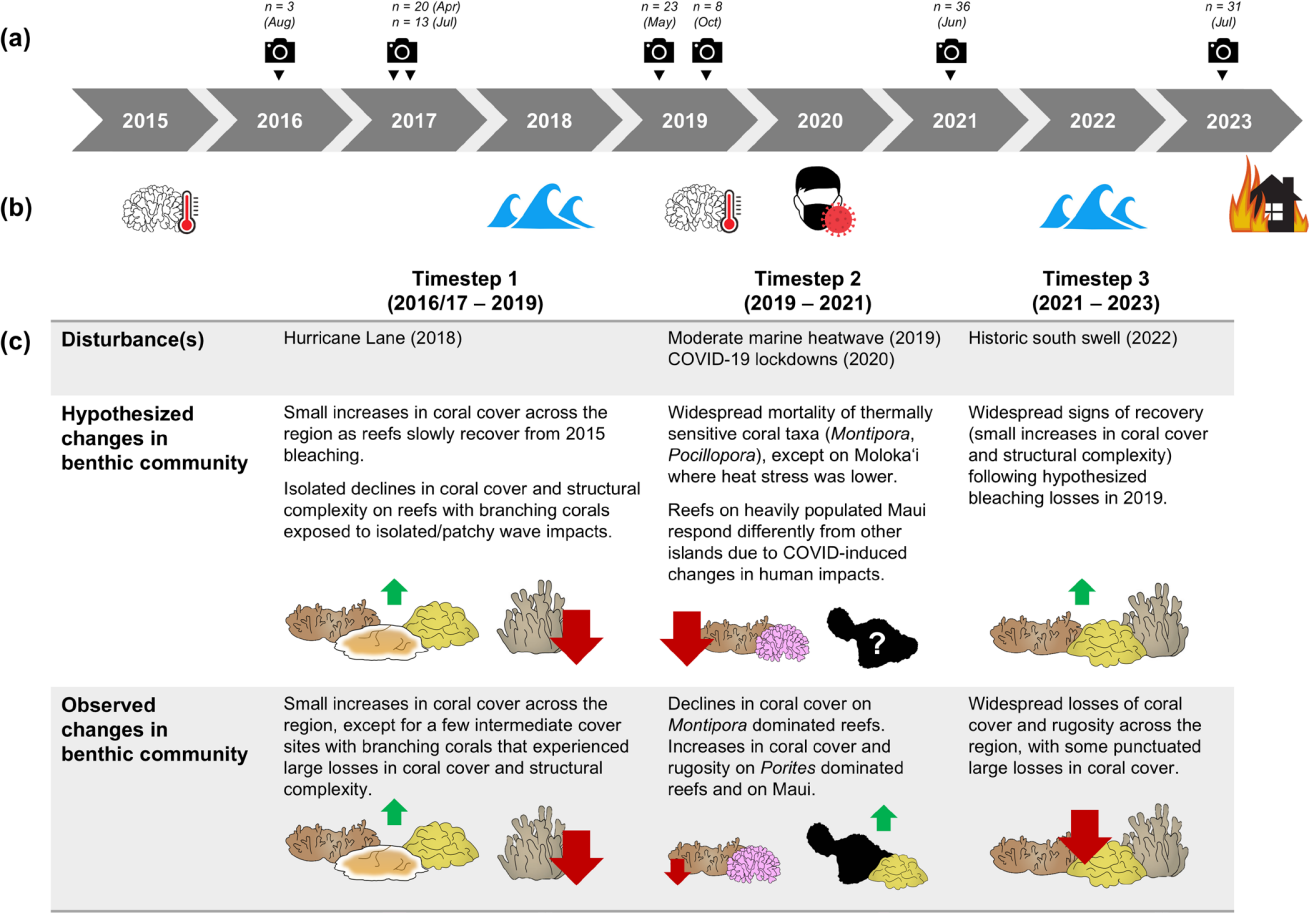
(a) A timeline of survey effort, with cameras representing field expeditions. The number of sites surveyed in each expedition is also noted. (b) A graphical representation of notable disturbance events in Maui Nui from 2015 to 2023. (c) Hypothesized patterns of coral cover and rugosity change in each timestep are compared with observed patterns of change. Observations were consistent with expectations for timestep 1, partially consistent for timestep 2, and not consistent for timestep 3. Vector graphics represent the following coral taxa, from left to right: *Montipora capitata* , *Montipora patula*, *Porites lobata* , *Porites compressa* , and *Pocillopora*.

We completed our first survey between July 2016 and July 2017 and resurveyed sites in 2019, 2021, and 2023 (Figure 1a). Most sites were surveyed four times (*n* = 30), while a minority were surveyed only three times (*n* = 2) or twice (*n* = 4). Between 2016 and 2023, several moderate disturbance events impacted reefs in the region (Figure 1a). The first major bleaching event documented in Maui Nui occurred just before the start of our timeseries (2015), and a second bleaching event of similar magnitude occurred during our timeseries in 2019 (Couch et al. 2017; McCarthy, Winston Pomeroy, and Smith 2024; Winston et al. 2022; Yadav et al. 2023). Hurricane Lane narrowly missed Maui Nui in 2018 but still generated a large swell, while a historic south swell impacted the region in 2022 (Nugent et al. 2020; Osher 2022). Finally, changes in human behavior due to the COVID‐19 pandemic (i.e., lockdowns, temporary cessation of tourism) altered anthropogenic impacts on Maui Nui's reefs in 2020 (Weng et al. 2023). It is worth noting that the devastating 2023 West Maui wildfires occurred just after our 2023 surveys. Nearshore impacts from wildfire debris and runoff are just now beginning to emerge and are outside the scope of this study.

### 2.2 Large‐Area Imagery Surveys

We surveyed fixed long‐term monitoring sites using large‐area imagery, also known as Structure from Motion photogrammetry. This technology relies on parallax, the apparent displacement of an object when viewed from multiple directions, to generate composite 2D or 3D models of benthic environments from overlapping imagery (Edwards et al. 2023). Using large‐area imagery, contiguous swaths of reef (hundreds of m^2^) can be precisely relocated and imaged over multiple years. These timeseries allow researchers to track changes in structural complexity and successional patterns of entire benthic communities at high spatial and taxonomic resolution (Ferrari et al. 2016; Magel et al. 2019; Sandin et al. 2020).

Our large‐area imaging surveys followed the methods of Edwards et al. 2017. A diver recorded the GPS coordinates of a stainless‐steel stake or eyebolt embedded in the reef, which served as a permanent reference for site relocation. Each 10 × 10 m site was temporarily marked with six calibration tiles and four reference floats to aid in diver navigation. During the survey, one diver measured the depth of each calibration tile and placed four 0.5 m long scale bars within the site boundaries. The other diver swam with a custom camera rig 1.5 m over the benthos in a gridded pattern to produce high overlap between photos. The rig contained two Nikon DSLR cameras (D7000, except for the 2023 surveys, which utilized D780) in Ikelite underwater housings. One camera was equipped with a wide‐angle lens (18 mm for D7000, 24 mm for D780) while the other had a longer focal length (55 mm for D7000, 60 mm for D780) to capture more magnified images. Lens focal lengths were changed slightly in 2023 to ensure that D780 photos covered the same spatial footprint as the earlier D7000 photos. Both cameras used an automatic 1‐s interval timer, which resulted in roughly 5000 photos per site. The diver swam several meters beyond the core 10 × 10 m site to capture a buffer zone of additional imagery. Each survey took approximately 50 min to 1 h to complete.

### 2.3 Data Processing

After each field expedition, we generated a dense point cloud reconstruction (3D model) of each site using *Agisoft Metashape*. Imagery from both cameras was aligned, but only imagery from the wide‐angle camera was used to generate 3D models. The longer focal length images, which had higher resolution but a smaller spatial footprint than wide‐angle images, were used as a reference for species identification. We used *Viscore*, a custom ecological analysis and visualization software (Petrovic et al. 2014), to input depth and scale metadata and align 3D models of the same site from different years (Sandin et al. 2020). This co‐registration of models allowed us to analyze the same 10 × 10 m benthic community over time. One site with incomplete image overlap (Kaho'olawe 6) necessitated the use of a rectangular (12 × 5 m) plot instead.

Using aligned 3D models, we quantified three site‐level metrics of benthic community composition for each timepoint: (1) percent cover of benthic taxa, (2) landscape heterogeneity, and (3) structural complexity. A single coral reef ecologist (OM) manually identified benthic cover at 2500 stratified random points for each site (10 × 10 m plot). Each point was identified to the finest taxonomic resolution, and then all point IDs were aggregated into 15 categories chosen to reflect the dominant benthic taxa and functional groups in Hawai'i (Figure S3a). We interpolated this spatially explicit benthic data using a Voronoi tessellation (Figure S3b,c) to quantify landscape heterogeneity, defined here as the proportion of Voronoi polygon boundaries where two different taxa border each other (termed “unlike adjacencies”; Figure S4). Finally, we quantified structural complexity using linear rugosity and fractal dimension, which we derived from depth measurements using Viscore's Virtual Profile Gauge tool (McCarthy et al. 2022), which assesses the height of the substrate along virtual transects. See Section S1 for additional details of each workstream.

Our metrics of percent cover, landscape heterogeneity, and structural complexity are important indicators of coral reef structure, composition, and function. In brief, high percent cover of corals and calcifying algae is generally considered to be an indicator of reef health (Smith et al. 2016), while the proportion of specific taxa within each benthic community can provide insights into successional dynamics and dominant life history strategies. Landscape heterogeneity is a measure of the aggregation and patchiness of the benthos (Cushman, McGarigal, and Neel 2008; Wang, Blanchet, and Koper 2014). On reefs with a high proportion of unlike adjacencies between Voronoi polygons, benthic taxa are less aggregated and form smaller patches, creating more opportunities for interspecific interactions (Figure S4). Structural complexity, quantified here using linear rugosity and fractal dimension, measures the roughness of the reef surface. Much of this roughness is created by reef‐building corals, which provide habitat for reef fish and mobile invertebrates (McCarthy et al. 2022; Nash et al. 2013). A perfectly flat surface has a linear rugosity of 1, with higher rugosity values corresponding to more structurally complex surfaces.

### 2.4 Statistical Analysis

To analyze spatial and temporal trends in benthic community composition, we calculated the Bray–Curtis dissimilarity among all sites in all years. Our multivariate data consisted of (1) percent cover of 15 benthic classes, (2) landscape heterogeneity (the proportion of unlike adjacencies), and (3) structural complexity metrics (linear rugosity collected at 1 and 50 cm resolution and fractal dimension). For comparability with percent cover data, we standardized landscape heterogeneity and structural complexity metrics to range from 0 to 1. We used non‐metric multidimensional scaling (NMDS) to visualize benthic community composition for all islands and years together in multidimensional space using the vegan package (Oksanen et al. 2022). Next, we analyzed the same multivariate benthic data with a permutational multivariate analysis of variance (PERMANOVA) to identify significant drivers of benthic community variation. These drivers included island (fixed effect; Kaho'olawe, Lāna'i, Maui, and Moloka'i), depth (m), wave power (max monthly mean, kW/m), sea surface temperature (standard deviation, °C), chlorophyll‐a (mean, mg/m^3^), surface irradiance (mean, mol m^−2^ day^−1^), sediment export (tons/ha/year), and effluent (gal/kg^2^/day) (Figure S6, Table S1). Environmental variables represent long‐term average conditions for our sites and were sourced from the Ocean Tipping Points project (Falinski 2016; Li et al. 2016; Wedding et al. 2018; Whittier and El‐kadi 2014). None of the environmental variables had a correlation coefficient more extreme than 0.54 (Figure S7a). We used the LDM package (Hu and Satten 2023) to perform a repeated measures PERMANOVA to account for the non‐independence of resampling fixed sites.

To identify groups of sites with similar benthic community composition, we used the same Bray–Curtis dissimilarity matrix of benthic data from our NMDS to conduct a hierarchical cluster analysis. We used the complete linkage method within the hclust function in R (R Core Team 2023). Our observations from the field led us to believe that three ecologically distinct community types exist, which was supported by the hierarchical clustering dendrogram (Figure S8a). To explore the robustness of our results to clustering method, we also implemented clustering using Ward's method (Figure S8b). While a few sites changed position, the same three ecological groupings were achieved regardless of the clustering method employed. In addition, we used analysis of similarities (ANOSIM) to test whether dissimilarity was greater between or within groups, first using island and then using cluster as the grouping factor. We calculated each ANOSIM using 9999 free permutations with the vegan package (Oksanen et al. 2022).

Our ANOSIM corroborated our observation that these clusters likely represent distinct benthic communities, so next we tested the hypothesis that reefs of the same community type would respond similarly to disturbance events regardless of their location within Maui Nui. We used mixed‐effects linear models to test whether the fixed factor of community type or island better described the following response variables: (1) change in coral cover, and (2) change in linear rugosity (1 cm resolution) in each timestep. We calculated each response variable as the monthly rate of change between each survey for each site to account for inconsistent durations between surveys. We included site as a random effect in our models and modeled variance using the varIdent function to account for heteroskedasticity (Pinheiro et al. 2018). We used Tukey's honest significance test to identify significant pairwise differences using the emmeans package (Lenth et al. 2023) and selected the best model using AIC.

Lastly, we used multiple regression with generalized additive models (GAMs) to identify which environmental variable(s) best explained patterns of change in coral cover and rugosity for each timestep of our monitoring program (2016/2017–2019, 2019–2021, and 2021–2023). We hypothesized that wave damage, bleaching, and sedimentation stress could have driven coral mortality during our timeseries (Figure 1c) and constructed a GAM to explore each hypothesis (Table S3). We also quantified maximum thermal stress at each site in 2015 to test whether bleaching impacts pre‐dating our monitoring could explain coral cover and rugosity change from 2017 to 2019. For our second and third timesteps, we tested the hypothesis that change in coral cover and rugosity in the preceding timestep would explain subsequent patterns of benthic change (via recovery dynamics, succession, or chronic degradation). Finally, we incorporated island and community type to account for the effect of local environmental factors and benthic community composition. For details of this analysis, see Sections S2 and S3. Code for all analyses described above is available on Dryad (McCarthy et al. 2025).

## 3 Results

### 3.1 Spatial Patterns of Benthic Community Composition

**FIGURE 2:**
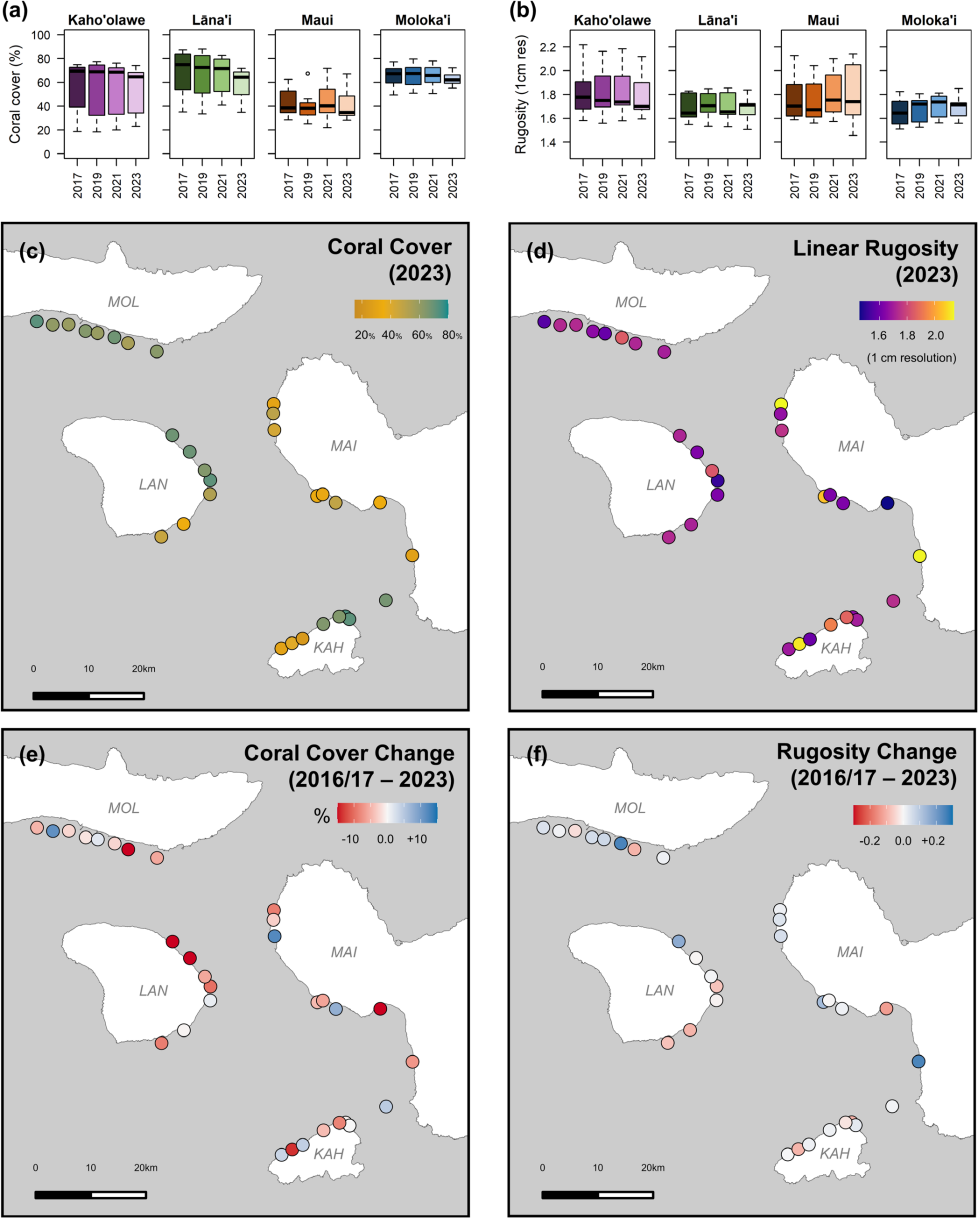
Boxplots show (a) percent coral cover and (b) fine‐scale linear rugosity (1 cm resolution) for each island in each year. Maps show site‐level values of (c) coral cover and (d) rugosity in Maui Nui in 2023 and net site‐level change in (e) coral cover and (f) rugosity over the full timeseries.

Throughout our timeseries, coral cover ranged from 18.36% (Kaho'olawe 6 in 2019) to 87.97% (Lāna'i 8 in 2019), with some sites dominated by encrusting and quasi‐branching *Montipora* corals and others by massive or branching *Porites* corals (Figure S1). As of 2023, coral cover was highest on Moloka'i (62.75% ± 1.92%; mean ± SE), followed by Lāna'i (58.26% ± 5.25%), Kaho'olawe (52.36% ± 8.05%), and Maui (40.82% ± 4.20%) (Figure 2a,c). Fine‐scale rugosity, which ranged from 1.45 to 2.22 (Figure 2b,d), was higher on Maui (1.80 ± 0.08) and Kaho'olawe (1.79 ± 0.07) and lower on Moloka'i (1.69 ± 0.03) and Lāna'i (1.68 ± 0.04).

**FIGURE 3:**
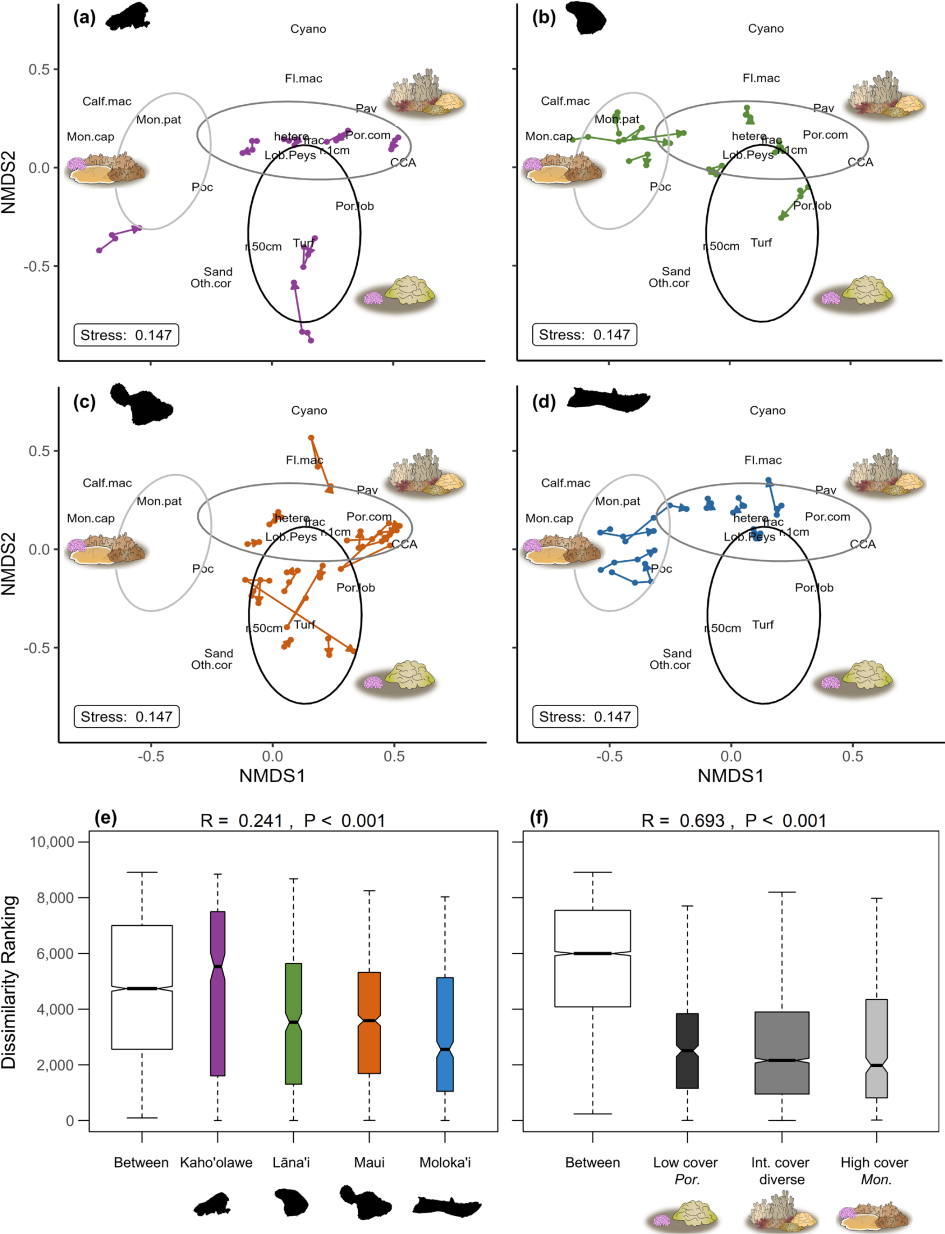
Non‐metric multidimensional scaling (NMDS) ordination of benthic community composition illustrates successional trajectories for reefs on (a) Kaho'olawe, (b) Lāna'i, (c) Maui, and (d) Moloka'i. ANOSIM dissimilarity ranks between and within groups are shown for (e) island and (f) community types identified through hierarchical clustering, which are graphically depicted in each NMDS with ellipses (light gray = high coral cover *Montipora* dominated reefs; dark gray = diverse intermediate coral cover reefs; black = low coral cover *Porites* dominated reefs). Text indicates benthic variables used in the ordination, including percent cover of various benthic taxa (see Figure S3), structural complexity (r.1 cm = rugosity at 1 cm resolution; r.50 cm = rugosity at 50 cm resolution; frac = fractal dimension), and landscape heterogeneity (hetero). Although visualized here using four separate panels, only one NMDS was conducted, which used benthic data from all islands and years.

We identified three distinct benthic communities via hierarchical clustering (Figure 3a–d), and our ANOSIM confirmed that benthic community variation was better explained by these three clusters (*R* = 0.6932) than by island (*R* = 0.2415; Figure 3e,f). The first of these three communities had the lowest coral cover (35.09% ± 3.20%) and was characterized by large amounts of limestone framework covered in turf algae (53.52% ± 2.50%), low landscape heterogeneity, and moderate structural complexity. The most common coral in these communities was massive *Porites lobata* (15.23% ± 6.32% of total benthic cover). Less abundant benthic taxa (i.e., *Leptastrea*, *Psammocora*, sponges, zoanthids, etc.) were also more common on these reefs compared to other reefs in the region, albeit still rare. The second community had intermediate coral cover (55.74% ± 3.24%), high landscape heterogeneity, and high structural complexity. These communities hosted a diverse assemblage of corals and algae, with relatively high cover of branching *Porites compressa* (11.45% ± 1.76%), encrusting *Pavona* (1.24% ± 0.37%), crustose coralline algae (CCA) (9.19% ± 2.37%), and fleshy macroalgae (2.49% ± 0.87%). Finally, the third community had the highest coral cover (67.82% ± 2.61%), limited open space, low landscape heterogeneity, and low structural complexity. These communities had high a percent cover of encrusting, plating, and quasi‐branching *Montipora capitata* (48.62% ± 2.07%) and encrusting *Montipora patula* (13.00% ± 4.17%). All summary statistics reported above are from 2023 but are representative of other years in our timeseries.

**FIGURE 4:**
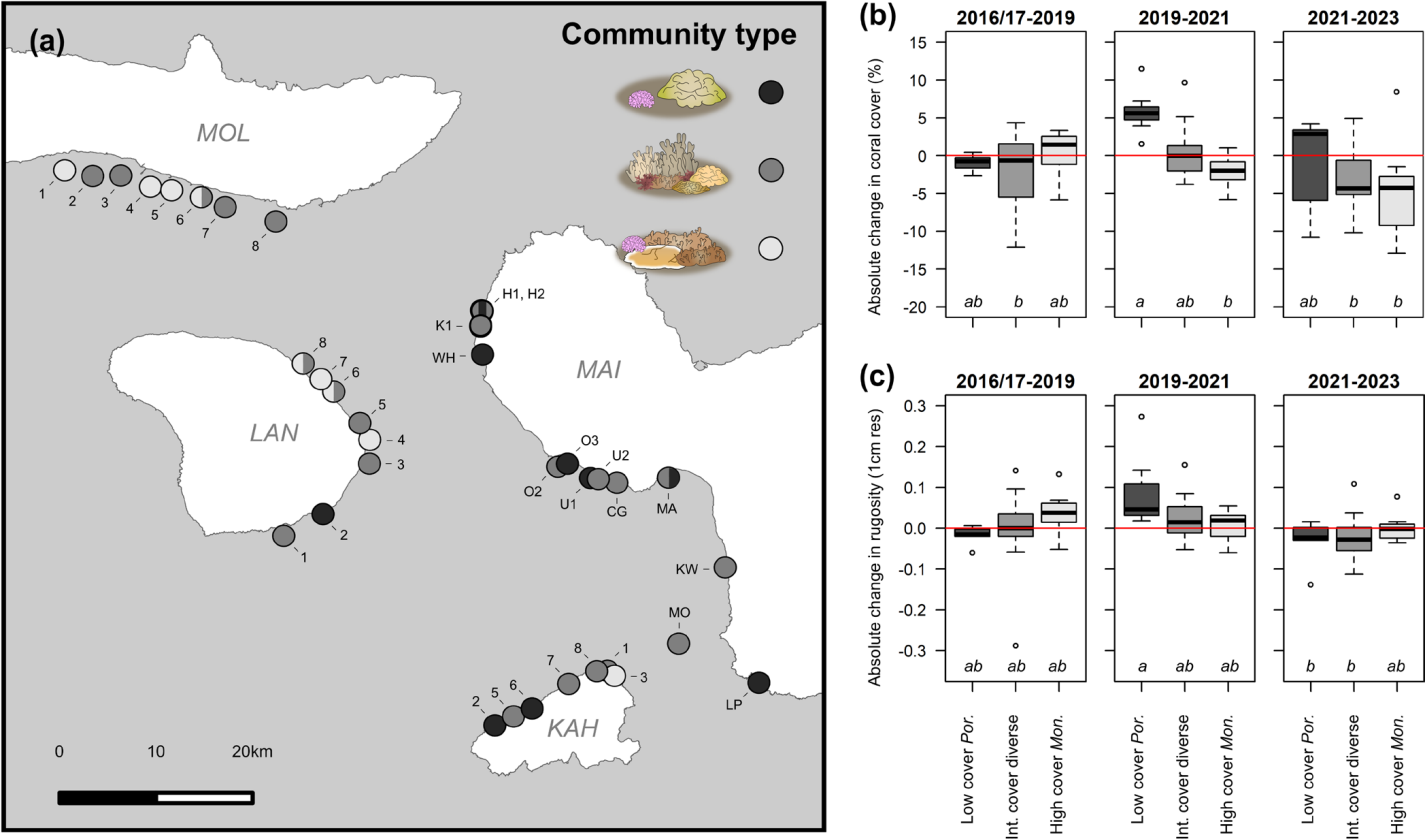
(a) The distribution of benthic community types across Maui Nui. Community types were as follows: Low coral cover *Porites* dominated reefs (black), diverse intermediate coral cover reefs (dark gray), and high coral cover *Montipora* dominated reefs (light gray). Points with multiple colors denote sites that shifted from one community type to another over the course of our timeseries. Only one site (H1) switched community types twice. Site abbreviations correspond to site names in Figure S1 and Figure S10. Change in (b) coral cover and (c) fine‐scale linear rugosity is shown for each timestep by community type. Letters denote significant differences between groups.

Intermediate cover reefs were widespread throughout the region, but the other two benthic communities were concentrated on specific islands (Figure 4a). Low coral cover *Porites* reefs were found most commonly on Maui and Kaho'olawe, while high coral cover *Montipora* reefs were found primarily on Moloka'i and Lāna'i. The average coral cover of each island reflects the spatial distribution of these community types. In addition, we performed a Mantel correlogram and found that benthic community composition was significantly spatially autocorrelated for sites within 5 km of each other, but not for sites located further apart (Figure S9).

The majority of benthic community variation (54.8%) could not be explained by island or the seven long‐term environmental variables analyzed here. Island explained the most variance (22.7%) of the variables we analyzed and was a significant predictor of benthic community composition (*p* < 0.001). Irradiance was also significant (*p* = 0.012) but explained a relatively small amount of variation (8.2%). None of the other environmental drivers displayed a significant relationship with benthic community composition (Table S1). In total, our seven environmental variables only explained 22.5% of benthic community variation.

### 3.2 Temporal Patterns of Benthic Community Composition

Compared to spatial variation, we observed less temporal variation on Maui Nui's reefs. Surveys from the same site in different years clustered tightly in multidimensional space (Figure 3, Figure S10), indicating that temporal variation within sites was smaller than spatial variation across sites. Furthermore, CRAMP sites located close to our large‐area imagery sites (*n* = 6) show evidence of temporal stability dating back to the early 2000s (Figure S2). Mean coral cover for the region was 55.75% ± 3.14% at the start of our timeseries in 2017 and 53.02% ± 2.87% at the end of our timeseries in 2023, although this apparent stasis masks notable change at individual sites (Figure 2e,f, Figure S1). In total, 14% of our sites transitioned from one community type to another at some point in our 6‐year timeseries (Figure 4a). All but one of these transitions were from higher to lower coral cover communities.

Diverse intermediate cover reefs showed the most stability in community composition: the NMDS successional trajectories of these sites tended to be short, compact, and non‐linear. Conversely, high‐cover *Montipora* sites showed more evidence of directional change over time, with most exhibiting a reduction in *Montipora* dominance (Figure 3, Figure S1). This community type experienced the largest loss in coral cover over the timeseries (Figure 4b) yet simultaneously experienced an increase in structural complexity (+0.07 ± 0.04; linear rugosity at 1 cm resolution; Figure 4c). The successional trajectories for low‐cover *Porites* sites were more variable, with some sites exhibiting stasis and others undergoing notable change over time (Figure 3, Figure S1).

Over the full 6‐year timeseries, coral cover increased on low‐cover *Porites* reefs (+4.89% ± 3.59%) and decreased on diverse intermediate cover reefs (−6.32% ± 2.32%) and high‐cover *Montipora* reefs (−8.81% ± 3.64%). Community types exhibited distinct trajectories of coral cover and rugosity, but these trajectories were not consistent over time (Figure 4b,c) or space (Figure S9b). The main effects of our mixed‐effects linear models (community type or island) were not significantly related to our response variables (change in coral cover or rugosity) in any of our models, but there was a significant interaction between our main effect and timestep in each model (Table S2). AIC indicated that community type was a better predictor of change in coral cover and rugosity than was island (Table S2).

## 4 Discussion

The Maui Nui region of Hawai'i represents a noteworthy bright spot of coral persistence in the Anthropocene (Cinner et al. 2016). The average coral cover we document across these 36 leeward forereef sites (55.75% ± 3.14% in 2017) is substantially higher than elsewhere in Hawai'i (24.1% ± 0.89% in 2012; Rodgers et al. 2015), the broader Pacific (21.3% ± 3.3% in 2016; Moritz et al. 2018), or the Caribbean (15.9% in 2019; Allen et al. 2021). Even accounting for the placement of our sites within known reef tracts with leeward exposure, the widespread occurrence of reefs with > 70% coral cover is remarkable given the degree of local human impacts and recent bleaching events in the region (Brown et al. 2008; Dailer et al. 2010; Jokiel, Cox, and Crosby 1995; Minton et al. 2022; Vermeij et al. 2008; Wedding et al. 2018). Here we interpret spatial and temporal patterns of variation and identify implications for natural resource management.

### 4.1 Spatial Patterns of Benthic Community Composition

We observed notable spatial variability in community composition (Figures 3 and 4) despite the fact that (1) coral reef benthic communities in Hawai'i generally have low diversity and are dominated by generalist taxa (Grigg 1983; Rooney et al. 2008), (2) our study was confined to sheltered leeward coastlines that experience similar oceanographic conditions (Shultz et al. 2004; Wedding et al. 2018; Figure S6, Table S1), and (3) the islands of Maui Nui share a common geologic history (Faichney et al. 2011; Field et al. 2019; Fletcher et al. 2008). Still, each island in Maui Nui has a unique history of colonization, land use, human impact, and environmental degradation (Brown et al. 2008; Jokiel, Cox, and Crosby 1995; Maynard et al. 2019; Minton et al. 2022; Ross, Rodgers, and Jokiel 2010). High variation in benthic community composition among islands and low variation within islands would suggest that island‐level regimes of human or land‐based impacts (i.e., sedimentation, wastewater pollution, and urban runoff) strongly shape coral reef condition (Gove et al. 2023). This understanding aligns with “ridge to reef” management in Hawai'i, where land‐based impacts on coral reefs are managed at the level of individual watersheds, or ahupua'a (Delevaux et al. 2018; Winter et al. 2020). On Maui in particular, localized impacts such as overfishing (Donovan et al. 2023; Williams et al. 2016), sediment runoff (Wedding et al. 2018), elevated nutrients (Dailer et al. 2010), invasive algae blooms (Smith, Hunter, and Smith 2002), and wastewater pollution (Winston et al. 2022) have been identified as chronic drivers of reef degradation (Rodgers et al. 2015; Sparks et al. 2015). These factors are almost certainly responsible for the lower coral cover we observed on Maui, given the lack of geologic and oceanographic differences between Maui and neighboring islands. However, while the effect of island was significant, the amount of variation it explained in our PERMANOVA was minor (22.72%), a finding corroborated by the low *R* value in our ANOSIM by island (*R* = 0.2415).

Similarly, the seven environmental drivers we considered, which represent long‐term average conditions for these reefs, collectively explained only 22.45% of the variation in benthic community structure (Table S1). This is due in part to our study design, where we controlled for wind exposure and depth over a small geographic area. High residual variation in community composition after controlling for environmental drivers is not unusual and has been documented in coral reefs (Gove et al. 2013; Murdoch and Aronson 1999; Sandin et al. 2022), wetlands (Reindl et al. 2023), mangrove forests (Guerra‐Castro and Cruz‐Motta 2018), and seagrass beds (Turner et al. 1999). In certain instances, the poor predictive power of environmental drivers has been attributed to anthropogenic disruption of key ecosystem processes (e.g., overfishing reducing herbivory), which can diminish or disrupt expected relationships between benthic communities and oceanographic forcings (Donovan et al. 2023; Williams et al. 2015, 2019). However, controlling for island (our proxy for local human impacts) should account for this decoupling. Instead, we found considerable spatial variation in benthic community composition that was not explained by oceanographic drivers or island (54.83% of variation).

The fine‐scale heterogeneity we observe could be driven by biotic processes not quantified here, such as recruitment, corallivory, herbivory, or competition. These processes have been shown to produce scale‐dependent feedbacks that lead to regular pattern formation in ecosystems (Rietkerk and van de Koppel 2008). For example, the density of reproductive adults has been shown to impact both recruit dispersal (Field et al. 2019; Pedersen et al. 2019) and survival (Marhaver et al. 2017), thereby influencing the spatial aggregation of conspecifics in subsequent generations (Pedersen et al. 2019; Sandin et al. 2022). Aggregations of corallivores, most notably crown of thorns starfish (*Acanthaster plancii*), can also contribute to the patchiness of benthic landscapes through spatially concentrated coral predation (Carlot et al. 2020; Houk et al. 2014). At low to moderate densities, selective feeding by *A. plancii* can contribute to landscape heterogeneity and promote diversity by preventing the dominance of competitive taxa (Colgan 1987), a phenomenon that has been documented previously on Maui (DAR 2006; Sparks et al. 2015). Similarly, the behavior and dispersal abilities of important reef herbivores (e.g., urchins, parrotfish, surgeonfish, etc.) have been found to differentially impact the composition of algal communities from the scale of centimeters to kilometers (Brandl and Bellwood 2016; Edwards et al. 2013; Sandin and McNamara 2012; Streit, Cumming, and Bellwood 2019), including in Maui Nui (Vermeij et al. 2010; Williams et al. 2016). This in turn shapes the competitive dynamics between corals and algae, which constantly compete for limited benthic space (Barott et al. 2012; George et al. 2021; Smith, Hunter, and Smith 2010). The arrangement and morphology of sessile competitors can shape the outcome of competition (Brito‐Millán et al. 2019; George et al. 2021; Rogers 1993) and lead to spatial self‐organization of taxa at larger scales (Rietkerk and van de Koppel 2008).

Environmental conditions interact with species morphology and life‐history traits to create spatial gradients in community structure (Ferrari et al. 2016; Murdoch and Aronson 1999; Sandin et al. 2022; Storlazzi et al. 2005; Wiens 1989). For example, coral taxa with compact growth forms and high skeletal densities tend to dominate habitats with more wave exposure, while sheltered reefs tend to support higher cover of fast‐growing yet delicate plating and branching corals (Dollar 1982; Donovan et al. 2018; Fletcher et al. 2008; Rodgers et al. 2015). When herbivory is viewed as a type of disturbance, a similar pattern of relative dominance can be seen in algae: on herbivore‐dominated reefs, less palatable algae such as CCA dominate, whereas fleshy algae and turf dominate on reefs with less herbivory (Kelly et al. 2017; Smith, Smith, and Hunter 2001). Intermediate disturbance resets competitive dynamics and promotes the persistence of multiple taxa. Without periodic disturbance, competitive coral or algal taxa will outcompete their slower‐growing neighbors to dominate the benthos (Darling et al. 2012; Kayal et al. 2015; Rogers 1993; Smith, Hunter, and Smith 2010), theoretically proceeding to a successional endpoint or “climax community” (Gouezo et al. 2019; Maragos and Grigg 1974; Turner et al. 1998).

Climax communities are rare on coral reefs because frequent disturbances typically prevent a single taxa from sustaining dominance (Gouezo et al. 2019; Grigg 1983; Hughes and Connell 1999). In this context, the reef tracts of Moloka'i and Lāna'i are noteworthy for their exceptionally high cover and dominance of *Montipora* corals (Figures 3 and 4), which suggests that these reef tracts have not historically been exposed to severe disturbances. This assertion is supported by surveys predating our study: an assessment of bleaching mortality following a severe marine heatwave in 2015 documented low coral mortality in south Moloka'i and northeast Lāna'i, compared to moderate mortality in West Maui (Rosinski et al. 2017). Whether or not south Moloka'i and northeast Lāna'i's reefs qualify as climax communities, it is worth noting they may not represent a “resilient” or “optimal” ecosystem state. *Montipora* dominance has resulted in more homogenous reefscapes with fewer opportunities for interspecific interactions. Structural complexity was also lower on these reefs due to the dominance of encrusting growth forms, which provide less habitat for reef fish of different size classes (Friedlander et al. 2003; McCarthy et al. 2022; Richardson, Graham, and Hoey 2017). A lack of functional redundancy and habitat diversity could leave these high coral cover communities vulnerable to degradation, especially since the most abundant taxa on these reefs are among the most susceptible to bleaching (Jokiel and Brown 2004; McLachlan et al. 2021; Winston et al. 2022) and predation by *A. plancii* (Sparks et al. 2015).

### 4.2 Temporal Patterns of Benthic Community Composition

Coral cover and rugosity trajectories were inconsistent from year to year and highly variable within each island (Figure 2e,f, Figure S9b). Other studies have similarly documented stability in average coral cover at island scales but high variation in reef trajectories within islands (Gove et al. 2023; Rodgers et al. 2015; Sparks et al. 2015). This fine‐scale variation supports the hypothesis that a single reef tract is in fact a patchwork of successional stages created by localized impacts from both acute and chronic disturbance events (Dietzel et al. 2021; Moritz et al. 2021; Murdoch and Aronson 1999; Ross et al. 2012; Turner et al. 1998). Here, we suggest that community composition can serve as a useful indicator of successional stage and future reef trajectory, following the Biological Legacies Model of succession (Pulsford, Lindenmayer, and Driscoll 2016). Indeed, we found that community type was a better predictor of coral cover and rugosity change than was island, which supports the notion that biotic processes (i.e., the life history strategies of dominant organisms) are important drivers of benthic community trajectories within Maui Nui's reefs. A similar pattern has been noted in terrestrial systems for forests (Burrows et al. 2019; Franklin et al. 2000; Imai et al. 2014; Lu et al. 2020) and grasslands (Purves and Law 2002; Staniczenko et al. 2017). While our study only captures a snapshot of succession, which takes decades to fully manifest on coral reefs (Gouezo et al. 2019; Maragos and Grigg 1974), it provides a useful mid‐successional glimpse of benthic community dynamics that can inform active reef management. By comparing benthic trajectories across a shared time horizon that includes multiple disturbances, we can detect early signals of stability, resilience, and vulnerability that could correspond more broadly to particular successional stages.

The three community types we identify here each responded differently to disturbance events over our timeseries. Intermediate coral cover reefs exhibited the least temporal variation, which could potentially indicate a stabilizing effect of species diversity, high interspecific competition, or frequent moderate disturbance in preventing any one taxon from gaining dominance (Connell 1978; Mouillot et al. 2013; Rogers 1993). In contrast, high‐cover *Montipora* reefs exhibited the strongest signs of directional succession, shifting away from *Montipora* dominance toward a more diverse coral community in response to several moderate disturbance events (Figure 3, Figure S1). It is more feasible for these reefs to experience large declines in cover compared to large increases, since the high density of *Montipora* and limited free space asymptotically limit further *Montipora* growth (George et al. 2021; Gouezo et al. 2019; Rogers 1993). A high density of conspecifics has also been shown to promote disease transmission, which could also limit coral growth (Aeby et al. 2011). Furthermore, it is possible that the lack of severe bleaching events prior to 2015 enabled the persistence of sensitive haplotypes within these climax communities and that multiple disturbances from 2017 to 2023 caused coral cover to decline via the loss of sensitive haplotypes (Burgess et al. 2021).

While coral cover on *Montipora*‐dominated reefs declined, coral cover increased on low‐cover, *Porites*‐dominated reefs. According to our PERMANOVA results, *Porites*‐dominated reefs were characterized by slightly higher sedimentation and nutrient effluent (Figure S10) and were more commonly found on the island of Maui (Figure 4). Thus, it seems likely that chronic human impacts on these reefs have favored the survival of stress‐tolerant corals, a finding supported by past studies of reef degradation in Maui (Ross, Rodgers, and Jokiel 2010; Ross et al. 2012). *P. lobata* was primarily responsible for the surprising increase in coral cover we observed on these reefs following the 2019 bleaching event (Figure 4b, Figure S12). This post‐bleaching increase could be due to coral acclimatization and selection for thermal tolerance following exposure to heat stress in 2015 (McCarthy, Winston Pomeroy, and Smith 2024), which would better position these communities to persist through future thermal stress as well. However, it is difficult to identify the impact of 2019 bleaching on coral trajectories because COVID‐19 could have caused a variety of confounding short‐term impacts. For example, reef fish biomass temporarily increased at Molokini, a popular dive and snorkel destination offshore of Maui, during the cessation of tourism in 2020 (Weng et al. 2023), which could have temporarily increased herbivory and boosted the competitive advantage of corals and CCA over fleshy algae (Smith, Smith, and Hunter 2001). In addition, a reduction in tourism could have reduced wastewater volume on Maui, temporarily relieving a source of chronic stress for corals (Gove et al. 2023). Yet, economic hardship and reduced oversight triggered by COVID lockdowns could have increased poaching within marine protected areas, as has been documented elsewhere (Coll 2020; King, Adhuri, and Clifton 2022; Phua et al. 2021), which could have reduced herbivory and boosted the competitive advantage of fleshy algae (Smith, Smith, and Hunter 2001). Thus, the impacts of COVID‐19 on marine conservation are likely multifaceted (Coll 2020), and it is currently unclear whether the pandemic represented a net positive or negative for Maui Nui's reefs.

Change in coral cover and rugosity in Maui Nui was best explained by wave and sedimentation impacts that occurred during our timeseries (Figures S7–S9), more so than marine heatwaves or COVID‐19. Storms represent a frequent source of disturbance for nearshore benthic communities, both via wave action (which can cause coral breakage and sediment resuspension; Minton et al. 2022; Storlazzi et al. 2005) and precipitation (which produces runoff that deposits new terrigenous sediment; Stamski and Field 2006). Sedimentation is a chronic stressor on nearshore environments across Maui Nui (Brown et al. 2008; Jokiel, Cox, and Crosby 1995; Maynard et al. 2019; Minton et al. 2022) and can reduce coral growth (by requiring corals to invest energy in increased mucous production to shed sediment) and recruitment (by covering suitable settlement substrate; Jokiel et al. 2014). Chronic stressors elevate already high levels of background mortality driven by intense competition among benthic organisms (Hughes and Connell 1999; Ross, Rodgers, and Jokiel 2010) and may better explain community resistance and recovery over time than acute stressors, both in Hawai'i (Gove et al. 2023; Rodgers et al. 2015; Ross, Rodgers, and Jokiel 2010) and elsewhere (Connell 1997; Cresswell et al. 2023). This relationship between chronic stress and ecosystem function supports the notion that reef condition can be improved through land‐based management actions that seek to reduce localized sources of chronic stress, specifically sedimentation and wastewater runoff (Anthony et al. 2015; Cinner et al. 2018; Gove et al. 2023; Obura et al. 2019).

## 5 Conclusions

By resurveying fixed sites with large‐area imagery, we were able to precisely track fine‐scale spatial and temporal changes in benthic community composition (Figure S5). This approach enabled us to characterize multiple aspects of ecosystem condition over time, including community composition, habitat structural complexity, and the spatial arrangement and interactions of benthic organisms. Importantly, large‐area imagery allowed us to capture a visual record of change across entire reefscapes, rather than isolated photo‐quadrats, and archive reef condition to support additional inquiry and hypothesis testing in the future. These data complement existing long‐term monitoring data from the state of Hawai'i (Brown et al. 2008; Sparks et al. 2015) and reveal remarkably high coral cover and temporal stability on Maui Nui's leeward reefs (Figure S2).

Still, the considerable spatial variation we document here serves as an important reminder that no two reefs are the same. Management informed by successional theory that accounts for this spatial heterogeneity will be better positioned to predict change and identify locally relevant drivers of reef decline (Rogers et al. 2015). As an example, targeted fisheries regulations in Kahekili (West Maui) have helped increase herbivory to counteract the localized impact of elevated anthropogenic nutrients (Brown 2019; Kelly et al. 2017; Ross et al. 2012; Smith, Runcie, and Smith 2005; Williams et al. 2016). Similarly targeted management approaches should be deployed to address emerging threats, such as the 2023 West Maui wildfires, which are likely to have patchy impacts on nearshore habitats via runoff of ash, debris, and toxic compounds (Wall et al. 2023). Long‐term monitoring programs, which enable managers to incorporate information about local disturbance history and successional trajectories, are foundational to such management (Crisp, Tebbett, and Bellwood 2022; Hughes and Connell 1999; Rodgers et al. 2015; Smith et al. 2016). Maintaining capacity for spatially and taxonomically detailed long‐term monitoring is of critical importance in our current era of increased global change.

## Author Contributions

**Orion S. McCarthy:** conceptualization, data curation, formal analysis, funding acquisition, investigation, methodology, project administration, software, supervision, validation, visualization, writing – original draft, writing – review and editing. **Emily L. A. Kelly:** conceptualization, formal analysis, investigation, methodology, project administration, supervision, writing – review and editing. **Anela K. Akiona:** data curation, formal analysis, investigation, writing – review and editing. **Samantha M. Clements:** data curation, formal analysis, investigation, project administration, supervision, writing – review and editing. **Tatiana Martinez:** data curation, formal analysis, investigation, writing – review and editing. **Nicole E. Pedersen:** data curation, formal analysis, investigation, methodology, project administration, software, writing – review and editing. **Cole Peralto:** data curation, formal analysis, investigation, writing – review and editing. **Sarah L. Romero:** data curation, formal analysis, investigation, writing – review and editing. **Mitchell H. Smelser:** data curation, formal analysis, investigation, writing – review and editing. **Kristy Wong Stone:** data curation, formal analysis, investigation, writing – review and editing. **Russell T. Sparks:** conceptualization, formal analysis, investigation, resources, writing – review and editing. **Jennifer E. Smith:** conceptualization, formal analysis, funding acquisition, investigation, methodology, project administration, resources, supervision, writing – review and editing.

## Conflicts of Interest

The authors declare no conflicts of interest.

## Supporting information


Data S1.

## Data Availability

The data and code that support the findings of this study are openly available in Dryad at https://doi.org/10.5061/dryad.15dv41p6r. Survey images from 2019 are provided for Lanāi (https://doi.org/10.6075/J05D8QCS), Kaho'olawe (https://doi.org/10.6075/J0JM285X), and Moloka'i (https://doi.org/10.6075/J01N7ZPH). Due to large file sizes, additional files are available upon request.
