# Supplementary material for: Using Community Composition and Successional Theory to Guide Site‐Specific Coral Reef Management
Source: Glob Chang Biol. 2025 Jan 28;31(1):e70050. doi: 10.1111/gcb.70050 (PMC11774138; doi:10.1111/gcb.70050)
Supplement: Supplementary file 1 — Data S1. [file GCB-31-e70050-s001.zip › Supplemental Materials.pdf]

Supplemental Materials for *Using community composition and successional theory to guide site-specific coral reef management*

Orion S. McCarthy[1], Emily L. A. Kelly[2], Anela K. Akiona[1], Samantha M. Clements[1], Tatiana Martinez[3], Nicole E. Pedersen[1], Cole Peralto[3], Sarah L. Romero[1], Mitchell H. Smelser[1], Kristy Wong Stone[3], Russell T. Sparks[3], Jennifer E. Smith[1]

1. Scripps Institution of Oceanography, UC San Diego, La Jolla, CA 92037, USA
2. World Economic Forum, San Francisco, CA 94129, USA
3. Hawai'i Division of Aquatic Resources, Maui, HI 96793, USA

Corresponding author contact: omccarth@ucsd.edu, +1 (301) 602-7329

Supplemental Section 1: Methods for ecological data extraction

Quantifying percent cover using large-area imagery

To assess percent cover, we identified benthic taxa to the finest possible taxonomic resolution using Viscore's Virtual Point Intercept tool. This tool mimics *in situ* point intercept sampling by placing a user-defined number of stratified random points, here targeting a density of 25 points/m^2 within a single 10 x 10 m quadrat (~2500 points total) in the 3D reef model (Fox et al., 2019). Previous work has found this density of points to be 1) sensitive enough to reliably detect benthic taxa with >1% cover, 2) ecologically relevant for analyzing landscape heterogeneity based on the typical size of coral colonies and algal patches, and 3) not excessively time consuming to collect (McCarthy et al. 2022; Fig. S5).

Our use of a single large (100m^2) quadrat, rather than small discontinuous quadrats (typically 0.5 to 1m^2, for *in situ* monitoring) has a number of advantages. For one, the approach captures information about the arrangement of benthic features, enabling additional metrics such as landscape heterogeneity to be calculated directly from percent cover outputs (Fig. S4). In addition, structural metrics such as linear rugosity can be collected over the same large area, allowing two different components of the benthic community (percent cover and rugosity) to be compared for the same tract of reef (McCarthy et al., 2022). However, by using a single large quadrat, we do not have replication to calculate standard error for our percent cover estimates.

There are multiple potential approaches to address this issue of replication. One approach would be to simply state that the area sampled (100m^2) is sufficiently large enough for our point-based estimates of percent cover to be representative of the "true" percent cover across the landscape. Given that we assessed benthic cover at 2,500 points within each site (25 points/m^2), this survey effort is considerably higher than would be achieved using smaller discontinuous quadrats. Another approach would be to subsample percent cover data from the large 10 x 10 m quadrat to create replicate quadrats *a posteriori*. However, these quadrats would likely not represent independent replicates, given their proximity, and this approach would not make use of most of the percent cover data that researchers spent hours collecting. A third approach would be to repeatedly sample the same reef using a fixed 10 x 10 m quadrat, and then calculate the confidence of our percent cover estimate based on the variance of these repeated samples. While it would take hundreds to thousands of hours to assess percent cover from benthic imagery for the same 10 x 10 m site repeatedly, we can rely on simulations to accomplish this task instead.

Using R, we created an empty 10,000 x 10,000 cell raster, where each cell represented 1 x 1mm. Then we generated a population of coral colonies (represented as circles) where colony area was selected from a log normal distribution, which has been shown to accurately represent colony size distributions for coral populations (Bak & Meesters, 1998; Rodriguez et al., 2021). We placed a random number of coral

colonies in the raster and then calculated the *true* percent cover of corals in this raster. Then, to simulate stratified random sampling with 2,500 points, we aggregated the original raster into a 50 x 50 cell raster, so that each cell would correspond to 20 x 20 cm. We randomly sampled one 1 x 1mm cell within each aggregated cell to mimic Viscore's stratified random sampling approach, and coded each aggregated cell as either "coral" or "empty". We calculated the *estimated* percent coral cover of the simulated reef based on these aggregated cells, and then calculated the absolute difference between true and estimated percent cover. We repeated this process 10,000 times, each time simulating a new coral population. Finally, we calculated the 95% quantile of the absolute difference in percent cover estimates. We repeated this entire process for coral communities with different mean colony sizes, ranging from $e^{1.75}$ (approx. $5cm^2$) to $e^{4.75}$ (over $100cm^2$). Values were interpolated to produce a 95% confidence estimate of percent cover, visualized a function of true percent cover and coral colony mean size (Fig. S5).

Based on this simulation, we can confirm that a single $100m^2$ plot is capable of producing robust and consistent estimates of percent cover. The estimate of percent cover was within 1.8% of the true percent cover of the reef in 95% of simulations for most coral size classes. Error was greater for coral populations with smaller size frequency distributions and for reefs where true percent cover was near 50%. To contextualize our results, we plotted six sites from Maui with available percent cover and coral colony size frequency data, which allowed us to gauge our confidence in percent cover estimates for real-world sites. For Maui, percent cover estimates appear to be ±1.6 to 1.8% of true percent cover, using a single 10 x 10 m quadrat with 2,500 stratified random points. One site with higher coral cover and larger colonies (Molokini) was within ±1.2 to 1.6% of true percent cover.

Based on our simulation, when assessing change in cover over time within a fixed quadrat, we can reasonably conclude that an absolute change of >3.6% is highly likely to represent actual change in percent cover and not just sampling error. Furthermore, this threshold decreases substantially when true percent cover approaches 0% or 100%. These findings can be extended to percent cover estimates of other taxa as well, not just estimates of total coral cover.

<u>Quantifying landscape heterogeneity from benthic point intercept data</u>

The arrangement of features in a landscape can provide information about the biotic and abiotic forces that drive spatial patterning, including an ecosystem's history of disturbance (Dietzel et al., 2021), interspecific interactions (Barott et al., 2012; George et al., 2021; Williams et al., 2013), and successional trajectories (Turner et al., 1998). Metrics that quantify the shape and arrangement of habitat patches have been used for decades in terrestrial landscape ecology to study habitat fragmentation and land use change (Krummel et al., 1987; McGarigal & Cushman, 2002), population connectivity (Kindlmann & Burel, 2008), and the impact of disturbance events (Haire & McGarigal, 2009).

Over the last decade, landscape ecology metrics have been increasingly applied to seascapes as well (Wedding et al., 2011), including for marine spatial planning (Pittman et al., 2011). On a smaller scale, studies have recently made use of large-area imagery to study the aggregation of individual benthic organisms by applying metrics such as the variance to mean ratio (Edwards et al., 2017; Price et al., 2021). The data used to analyze the spatial patterning of taxa in marine systems include habitat maps derived from aerial or satellite surveys (large scale) to census-based assessments of individual sessile organisms (small scale). There is however a lack of approaches available to study the patch dynamics of benthic taxa at intermediate (i.e., patch-level) scales.

Point intercept data from percent cover surveys can be applied to bridge this gap. Historically, point intercept data has not been applicable for spatial pattern analysis because benthic cover is typically assessed using discontinuous quadrats or along a linear transect. However, using large-area images derived from photogrammetry (i.e., orthoprojections or photomosaics), percent cover can be assessed over a continuous extent using point intercept methods. If sufficient points are used in a percent cover survey, point intercept data can accurately represent the spatial arrangement of benthic taxa (Fig. S15). Compared

to census-based assessments of individuals, point intercept data can be collected quickly (McCarthy et al., 2022) which enables analysis of more and/or larger sites. More importantly, point intercept data doesn't require researchers to define what constitutes an "individual" organism, and instead facilitates analysis at the patch scale. This is noteworthy because it can be challenging to delineate "individual" organisms for coral species with high fusion/fission dynamics (i.e., *Porites compressa*) or for other benthic taxa (i.e., turf algae, crustose coralline algae). In addition, conducting spatial analyses at the "patch" scale may be more ecologically relevant for certain ecological questions (i.e., questions about how fish and other mobile organisms interact with the benthos) than the organism scale.

While point intercept data won't be suitable for analyzing all types of spatial pattern (especially at very fine-scales), ecological questions that involve habitat fragmentation or the patchiness of benthic community composition at the scale of 10s to 100s of meters may be well suited for this type of analysis. One such application is the study of succession and the response of the benthic community to disturbance. Following the intermediate disturbance hypothesis, we would expect species richness and diversity to peak at intermediate levels of disturbance where weedy, stress tolerant, and competitively dominant taxa can all coexist (Connell, 1978; Dollar, 1982; Grigg, 1983). Much like species diversity, the spatial aggregation of benthic classes should also exhibit characteristic patterns based on the frequency of disturbance or successional stage (Turner et al., 1998). For example, we would expect patches of benthic taxa to be smaller, more dispersed, and/or more fragmented on more diverse reefs where interspecific competition is high, and more aggregation on less diverse reefs, be they highly disturbed degraded reefs or climax communities with high coral cover.

Several studies have shown that landscape metrics are dependent on both the grain and extent of a spatial dataset (Cushman et al., 2008; Šímová & Gdulová, 2012; Turner et al., 1989), although some metrics are more dependent on scale than others. In this study, we calculated landscape heterogeneity by interpolating benthic point intercept data for each reef using a Voronoi tessellation (Fig. S3). From this tessellation, we calculated the proportion of Voronoi polygon boundaries where two different taxa border each other (termed "unlike adjacencies"; Fig. S4).

To test the scale dependence of this metric, we conducted a power analysis using four 10 x 10 m sites from Palmyra Atoll where coral colony boundaries had been previously traced. Using this data as a reference of "true" percent cover, we simulated stratified random sampling using point densities ranging from 1 to 500 points/m^2 (Fig. S15). We found that the number of patches, mean patch area, and proportion of unlike adjacencies were all scale dependent, but the rank order of sites remained consistent. In other words, the same site always had the highest proportion of unlike adjacencies, regardless of the number of points used to assess percent cover (Fig. S15). These results suggest that 25 points/m^2 (the current standard of data collection) would be a reasonable point density to use for landscape-level spatial patterning analyses (Fig. S5).

<u>Measuring structural complexity using large-area imagery</u>

We quantified structural complexity using linear rugosity and fractal dimension, which we derived from depth measurements collected using Viscore's Virtual Profile Gauge tool (McCarthy et al., 2022). This tool uses virtual rods to measure the height of the substrate along virtual transects, and the spacing between each rod determines the measurement resolution. Linear rugosity is calculated by dividing the length of the reef contour by the horizontal length of the transect. A perfectly flat surface has a linear rugosity of 1, with higher rugosity values corresponding to more structurally complex surfaces. Transects were oriented in the alongshore direction and spaced every 10 cm within the same 10 x 10 m plot used to assess benthic cover, and rugosity was averaged across transects to produce a single site-level value. We measured linear rugosity at two resolutions (1 cm and 50 cm) and calculated fractal dimension (D) as the rate of change in rugosity between those resolutions (McCarthy et al., 2022; Nash et al., 2013). Fine-scale rugosity (1 cm resolution) is indicative of coral-generated structure, while coarse-scale rugosity (50 cm resolution) is indicative of structure created by reef geomorphology (McCarthy et al., 2022).

Supplemental Section 2: Drivers of benthic change in each timestep

While we used long-term environmental averages in our PERMANOVA, our GAMs analysis sought to identify drivers of benthic change in each timestep, and thus necessitated timestep-specific data rather than long-term averages. We obtained site-level data for each timestep (2016/17–2019, 2019–2021, and 2021–2023) for three environmental variables: thermal stress (max degree heating weeks (°C/weeks); NOAA Coral Reef Watch 2020), wave height (90th percentile (m); Cheung 2021), and turbidity (mean of quarterly max (FNU); Dogliotti et al. 2015; Li et al. 2022). Turbidity data were not available for our first timestep, so for each site we also quantified the distance to the nearest stream as a proxy for sedimentation (Hawai'i Statewide GIS Program 2016), which we log transformed to reduce the influence of outliers. We also quantified maximum thermal stress at each site in 2015 to test the hypothesis that bleaching impacts pre-dating our monitoring were responsible for coral cover and rugosity change from 2017–2019. For our second and third timestep, we tested the hypothesis that change in coral cover and rugosity in the preceding timestep would explain subsequent patterns of benthic change (via recovery dynamics, succession, or chronic degradation). Finally, we also incorporated island and community type to account for the effect of local environmental factors and benthic community composition. To avoid issues related to multicollinearity, we didn't include environmental predictors with a correlation coefficient > 0.5 (Fig. S7), dropping the predictor believed to be less causally related to benthic community change. We used the package mgcv to implement our GAMs in R (Wood, 2011). We initially limited the basis dimension to k = 5, and decreased k if model results appeared to overfit the data. We selected the best models for coral cover and rugosity in each time step using AIC.

The first timestep (2016/17–2019) showed no significant changes in rugosity but a slight decrease in coral cover on diverse intermediate cover reefs (-1.93% ± 1.15%; Fig. 4b), best described in our GAMs by a combination of wave height, distance to the nearest stream, and island (adj. R^2 = 0.483; Fig. S11). During the second timestep (2019–2021), coral cover increased significantly on low cover *Porites* reefs (+5.85% ± 1.15%) and on Maui (+4.93% ± 1.71%), and decreased significantly on high cover *Montipora* reefs (-2.09% ± 0.75%; Fig. 4b). Rugosity also increased significantly on low cover *Porites* reefs (+0.08 ± 0.03) during this timestep. Coral cover and rugosity were more likely to decline on reefs with high turbidity that were proximate to streams (coral: adj. R^2 = 0.794; rugosity: adj. R^2 = 0.736; Fig. S12). Furthermore, reefs that experienced a decline in rugosity in the prior timestep (2016/17–2019) were more likely to see rugosity increase during the second timestep. In the final timestep (2021–2023), coral cover decreased significantly on diverse intermediate cover reefs (-4.60% ± 1.52%), high cover *Montipora* reefs (-4.68% ± 2.75%), and Lāna'i (-6.21% ± 1.84%; Fig. 4b). Rugosity decreased significantly during the final timestep at low cover *Porites* reefs (-0.03 ± 0.02) and diverse intermediate cover reefs (-0.02 ± 0.01; Fig. 4c). Declines in coral cover and rugosity during the final timestep were best explained by a combination of wave height, depth, and island (coral adj. R^2 = 0.325, rugosity adj. R^2 = 0.492; Fig. S13).

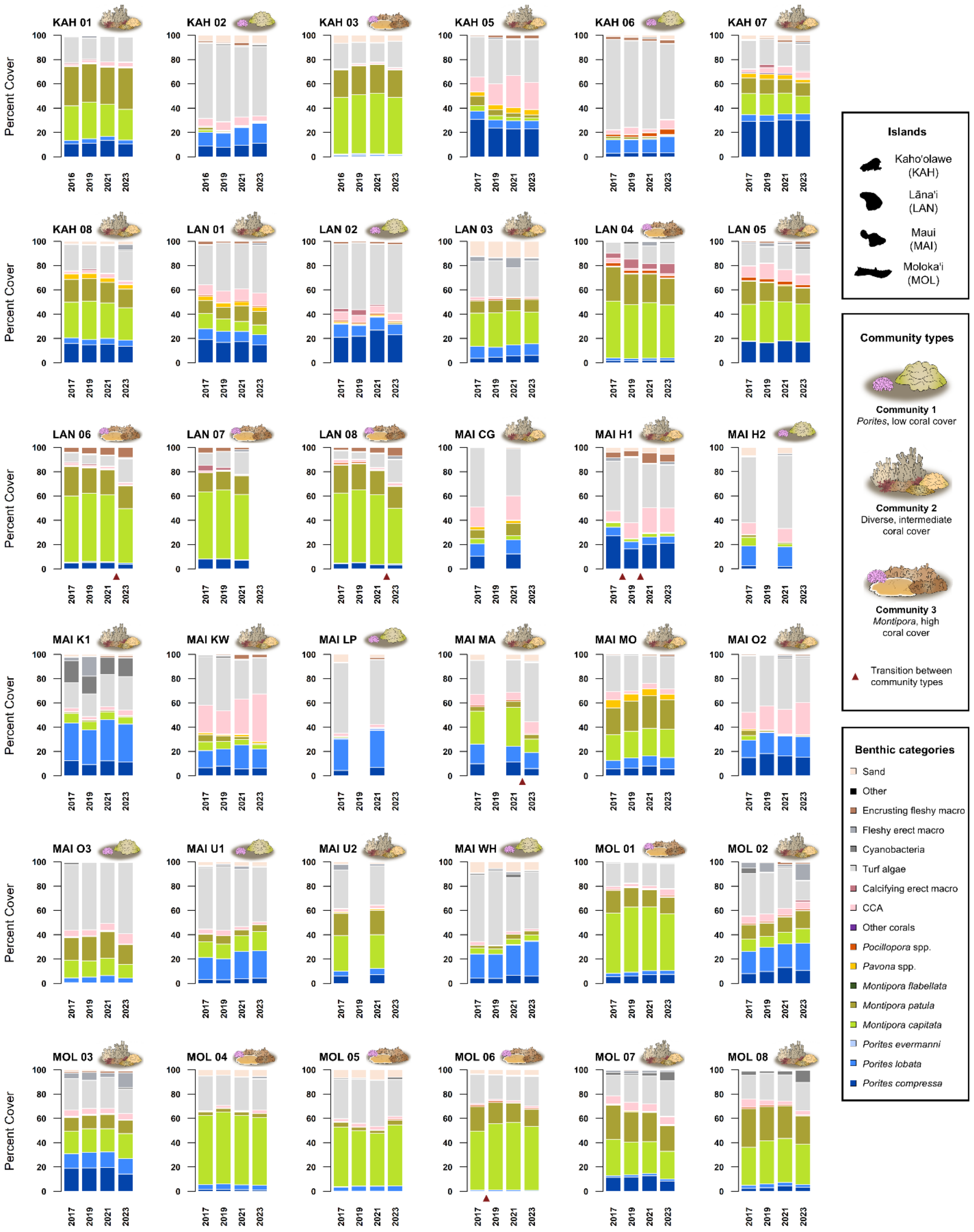

Figure S1. Benthic community composition for all sites in all years. Graphics denote each site's community type at the start of the timeseries. Red triangles denote timesteps where a site shifted from one community type to another. Site names correspond to the abbreviations used in Fig. 4a and Fig. S10.

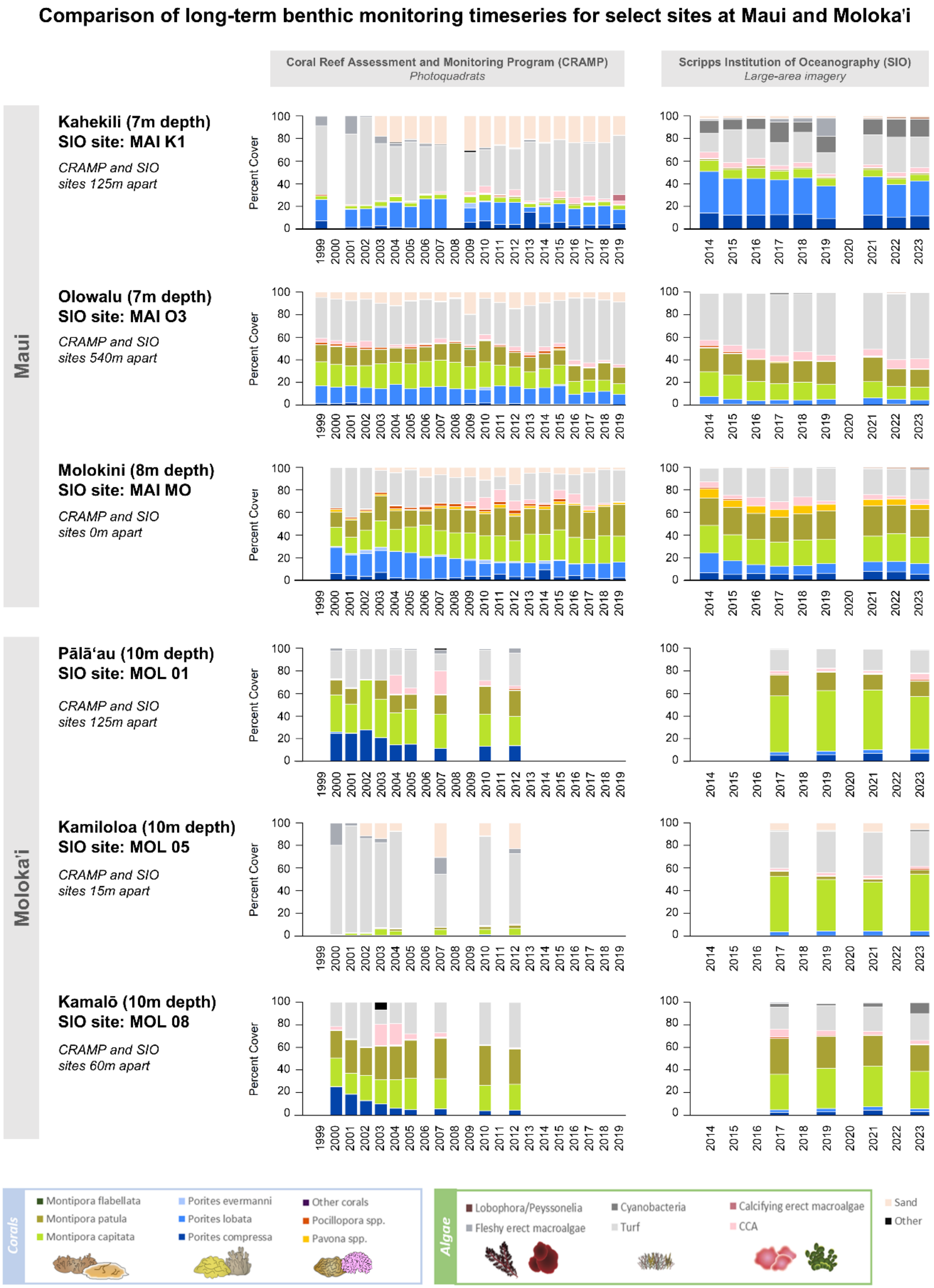

Figure S2. A comparison of long-term monitoring data collected at neighboring fixed long-term monitoring sites. SIO data was collected using large-area imagery while CRAMP data was collected using permanent photoquadrats. Colors denote benthic taxa.

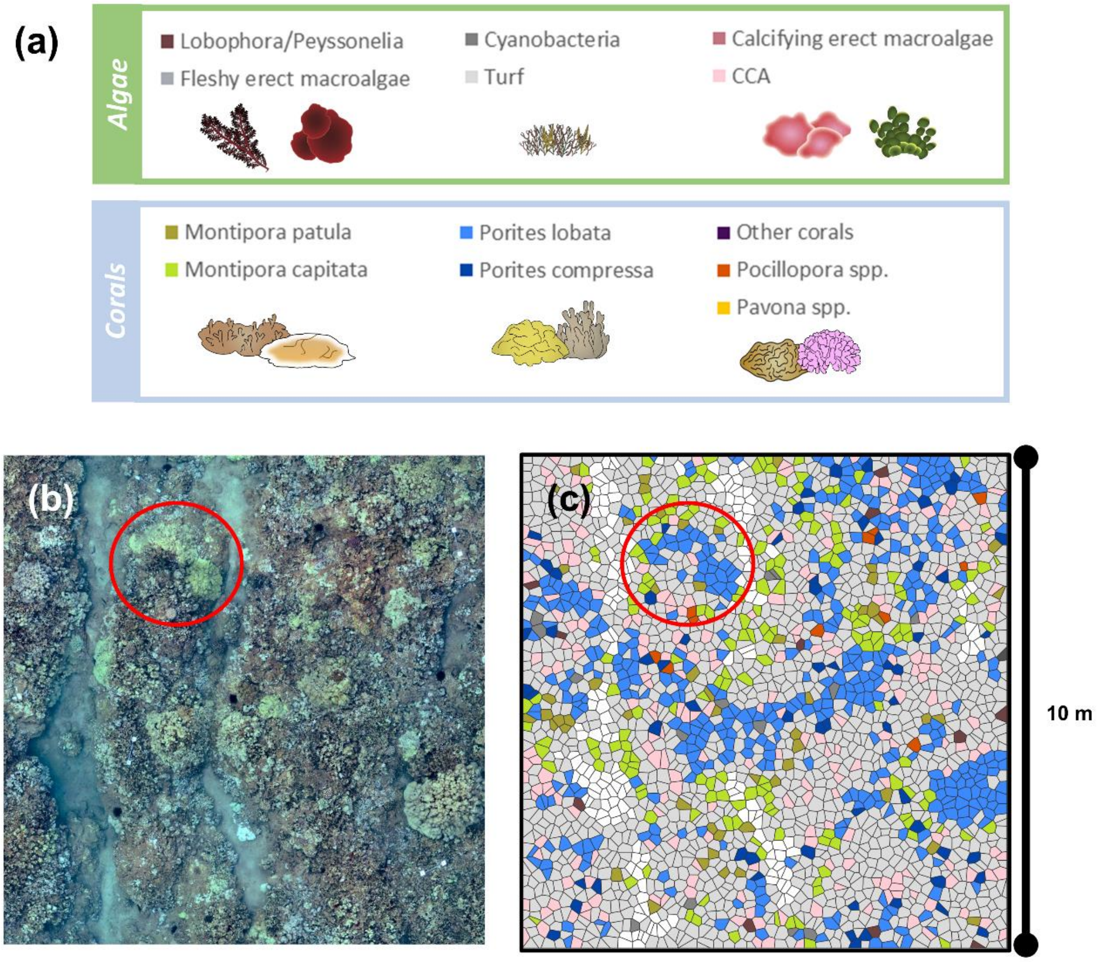

Figure S3. (a) We identified benthic cover to the finest taxonomic resolution at 2,500 stratified random points per 10 x 10 m site. Coral IDs were assigned to one of seven categories (*Montipora capitata, Montipora patula, Porites compressa, Porites lobata, Pocillopora* spp., *Pavona* spp., and other coral), while algae were assigned to one of six functional groups (turf, crustose coralline algae (CCA), calcifying erect macroalgae, fleshy erect macroalgae, fleshy encrusting macroalgae (*Lobophora* or *Peyssonnelia*), and cyanobacteria). Remaining points were categorized as either "sand" or "other", which mostly consisted of zoanthids, sponges or tunicates. (b) A top-down view of a 10 x 10 m site from Maui, compared with (c) benthic cover data from that site, interpolated via a Voronoi tessellation to form a continuous habitat map. The red circle in (b) and (c) correspond to the same large *Porites* colony. Use (a) as a legend to interpret the benthic categories in (c).

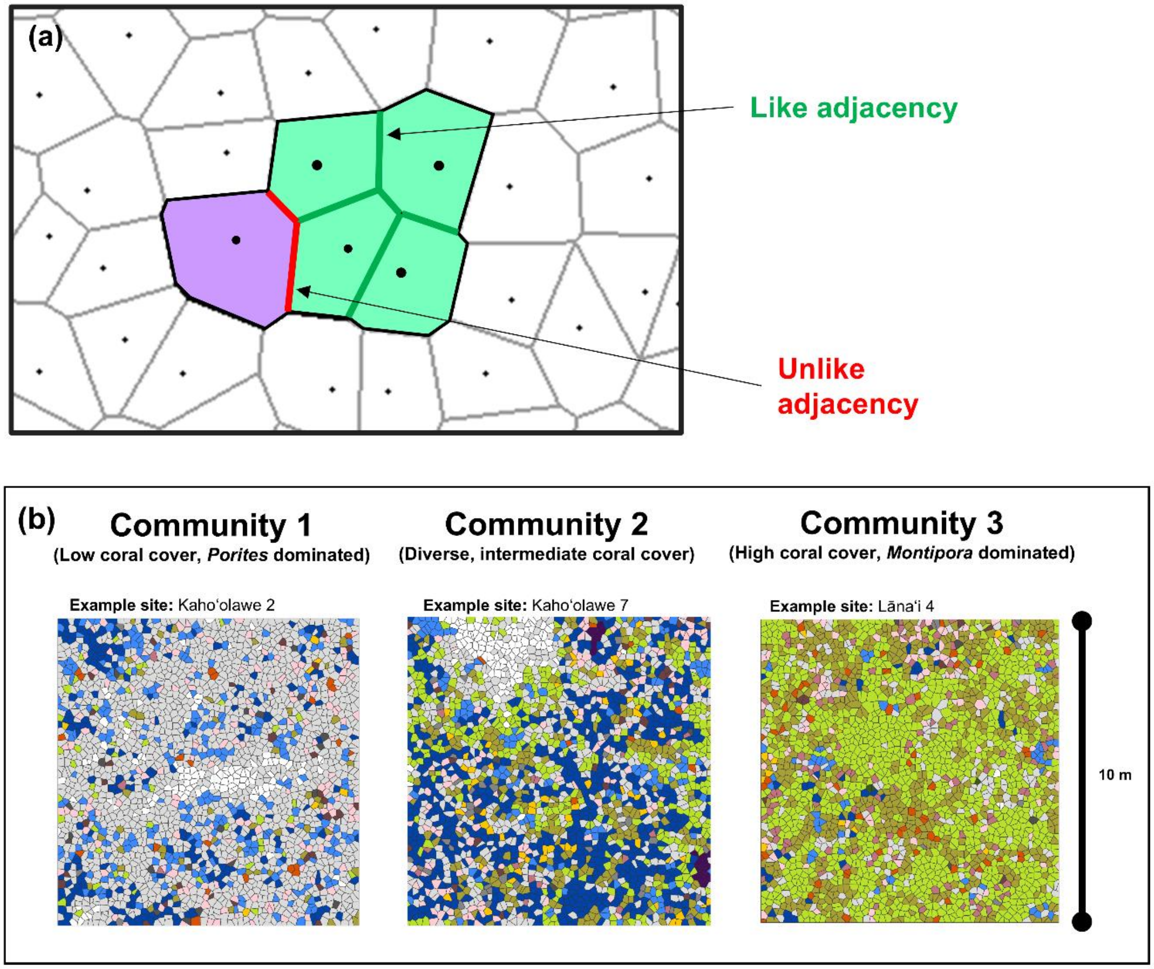

Figure S4. (a) Landscape heterogeneity is illustrated using a Voronoi tessellation. Points in (a) represent stratified random points used for benthic species ID. These point data have been interpolated via a Voronoi tessellation so that each polygon contains one point, and the area inside the polygon is closer to that point than to any other point in the dataset. Points can be considered "adjacent" if their polygons share a boundary. Adjacent points of the same benthic class are termed "like adjacencies", while adjacent points from different classes are termed "unlike adjacencies". We use the number of unlike adjacencies divided by the total number of adjacencies as our metric of landscape heterogeneity. (b) Representative Voronoi tessellations for each community type. Most adjacencies in communities 1 and 3 are like adjacencies, reflecting a more compact landscape with large contiguous patches. Community 2 has more unlike adjacencies and thus has smaller patches and more opportunities for interspecific interactions between taxa. Use Fig. S3 as a legend to interpret the benthic categories here.

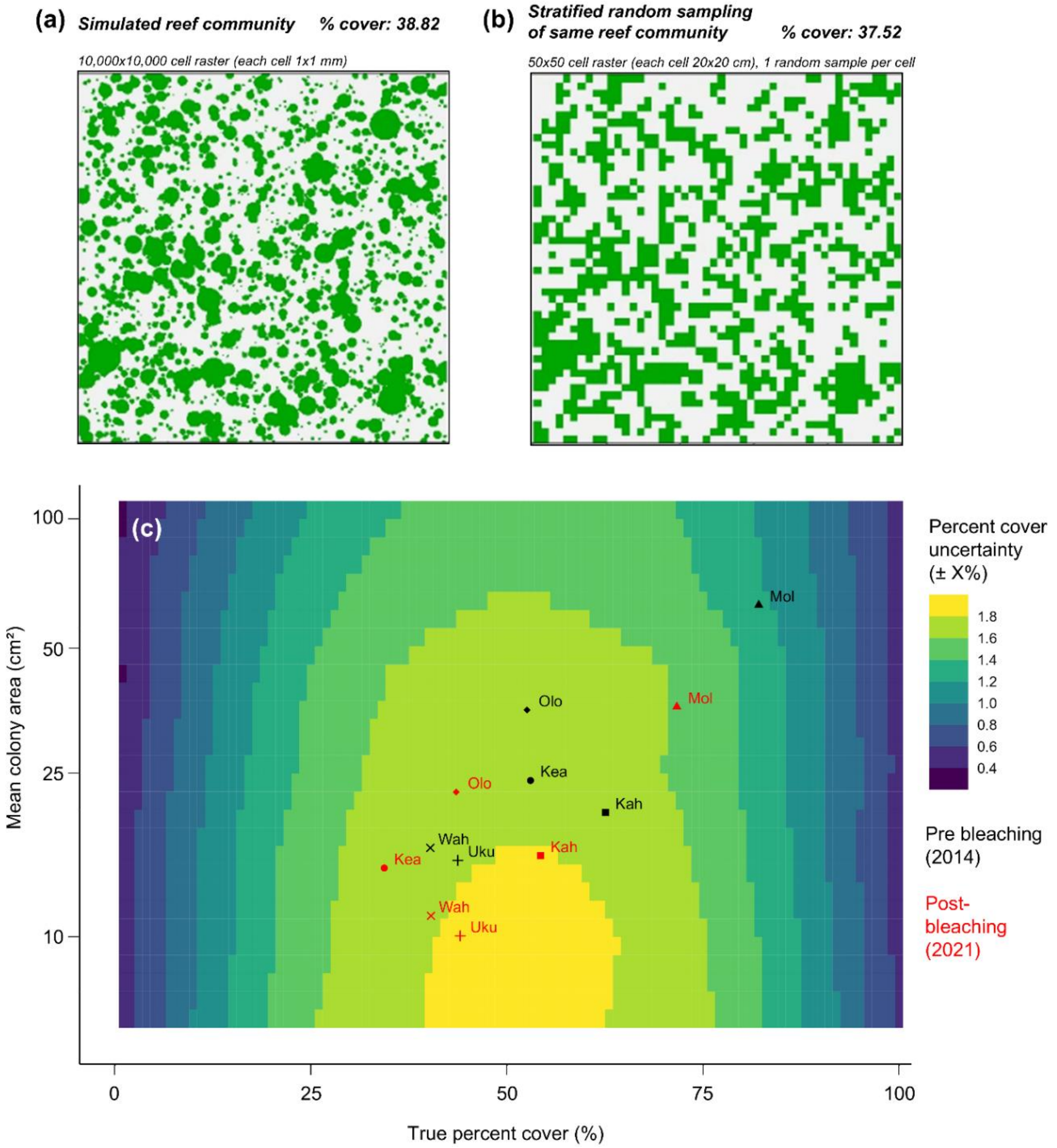

Figure S5. An example of (a) a simulated coral community where circles represent coral colonies, and (b) stratified random sampling of that same community using a 50x50 cell raster (2,500 points). The difference between estimated percent cover (via stratified random sampling) and true percent cover of the simulated community was calculated for 10,000 simulations, and repeated for various size frequency distributions of corals. (c) Colors indicate the difference between true and estimated percent cover (95% quantile of absolute difference) as a function of colony size frequency and total percent cover. Six sites from Maui with available percent cover and colony size frequency data are plotted, both from before bleaching (black) and after (red), to illustrate how confidence in percent cover estimates can vary as a function of the spatial and temporal dynamics of benthic communities. Site abbreviations are as follows: Kah = Kahekili, Kea = Keawakapu, Mol = Molokini, Olo = Olowalu, Uku = Ukumehame, and Wah = Wahikuli. Overall, our approach of using a single 10 x 10 m quadrat with 2,500 stratified random points appears to be a highly precise technique for assessing percent cover.

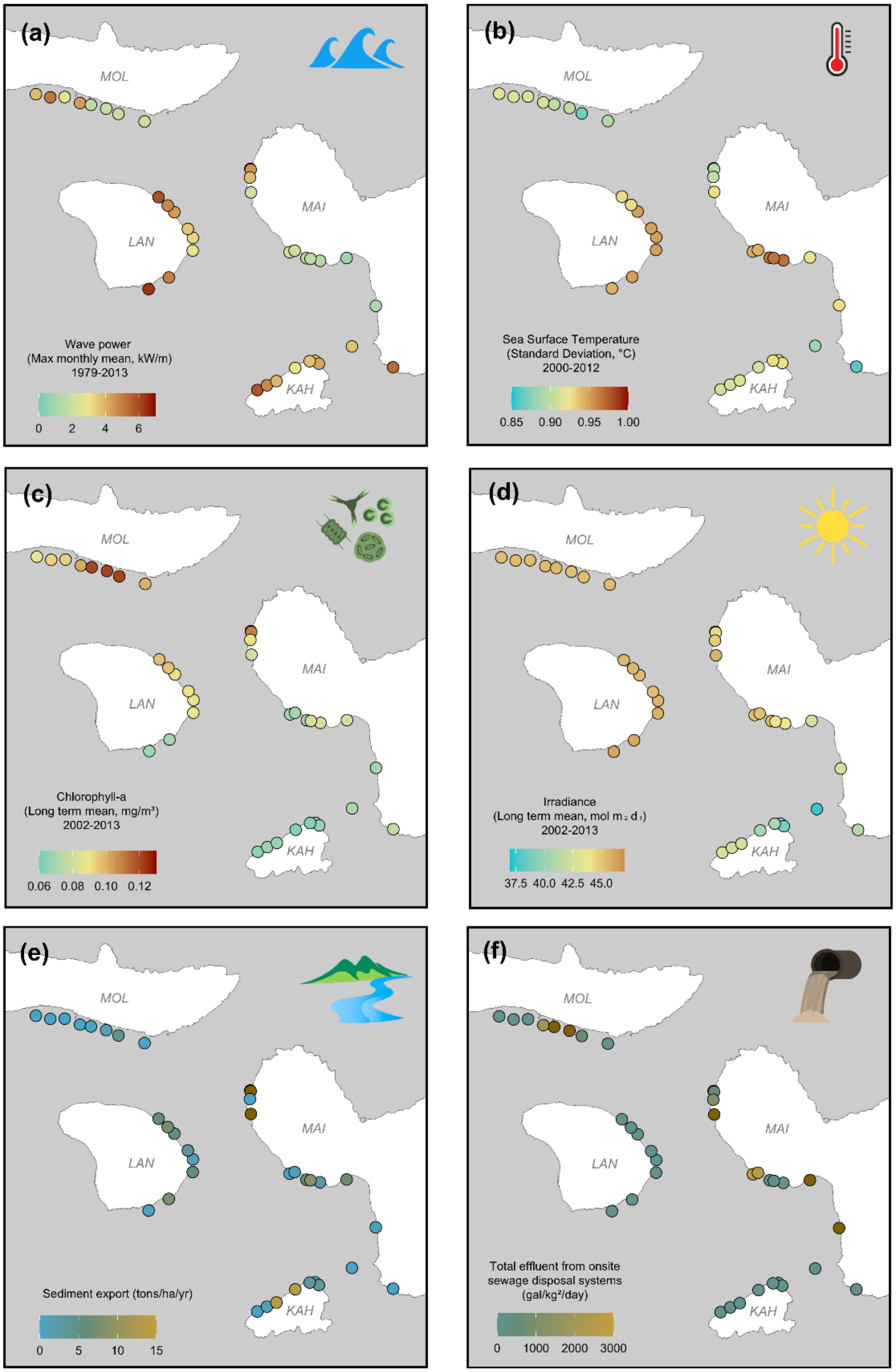

Figure S6. A map of environmental drivers used in the PERMANOVA. Data was obtained from the Ocean Tipping Points project (Wedding et al. 2018) and includes (a) wave power (max monthly mean, kW/m), (b) sea surface temperature (standard deviation, °C), (c) chlorophyll-a (mean, mg/m3), (d) surface irradiance (mean, mol m^{-2} d^{-1}), (e) sediment export (tons/ha/yr), (f) effluent (gal/kg^2/day). These data represent the long-term (mean) environmental conditions prior to our timeseries, rather than punctuated (max) conditions associated with disturbance during our timeseries.

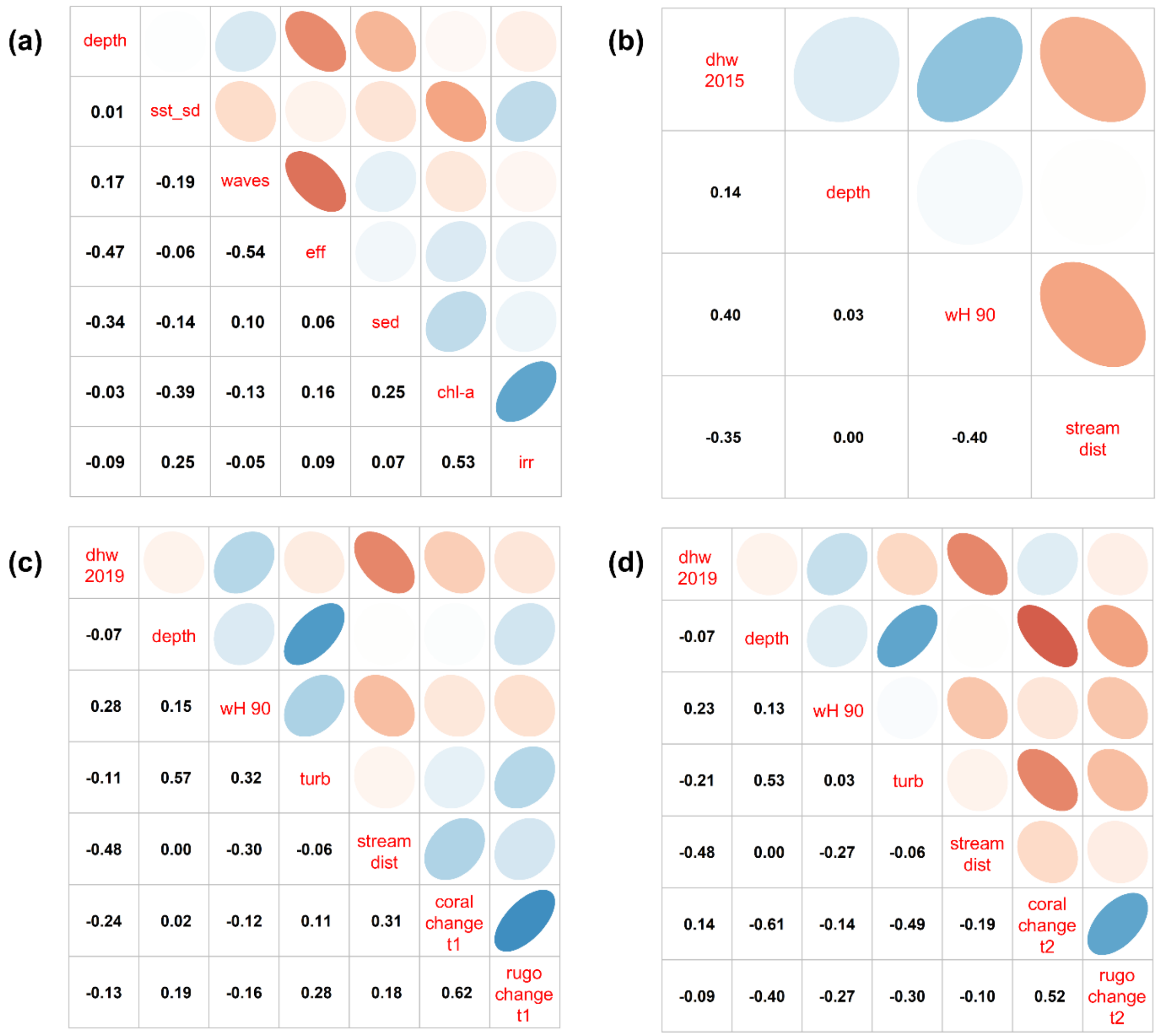

Figure S7. (a) Correlation plot for long-term environmental data sourced from Ocean Tipping Points. Correlation plots of environmental variables specific to each timestep are also shown for (b) 2016/17 to 2019, (c) 2019 to 2021, and (d) 2021 to 2023. Abbreviations are as follows: sst_sd = standard deviation of sea surface temperature, dhw = max degree heating weeks, wH 90 = 90th percentile of wave height, turb = mean of quarterly max turbidity, stream dist = distance to the nearest stream (log transformed), eff = total effluent, sed = sediment export, chl-a = chlorophyll-a, irr = irradiance.

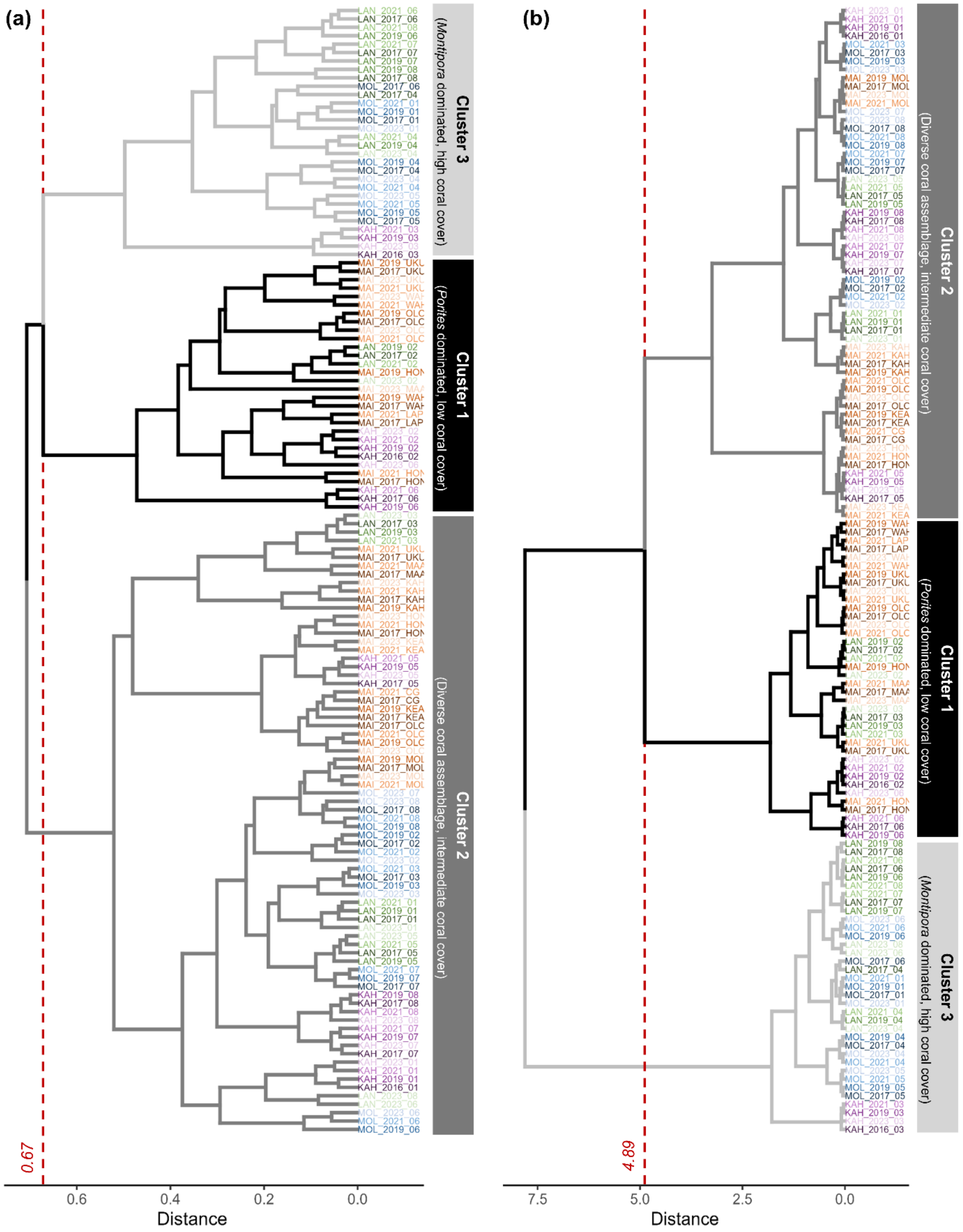

Figure S8. Hierarchical clustering of sites by benthic community composition visualized as a dendrogram using (a) the complete linkage method and (b) Ward's method. The complete linkages method was used for all analyses, but both clustering approaches identified the same three ecological groupings. Text color denotes island and sampling year (same color scheme as Fig. S10). The dotted line indicates where the dendrogram was split into three clusters.

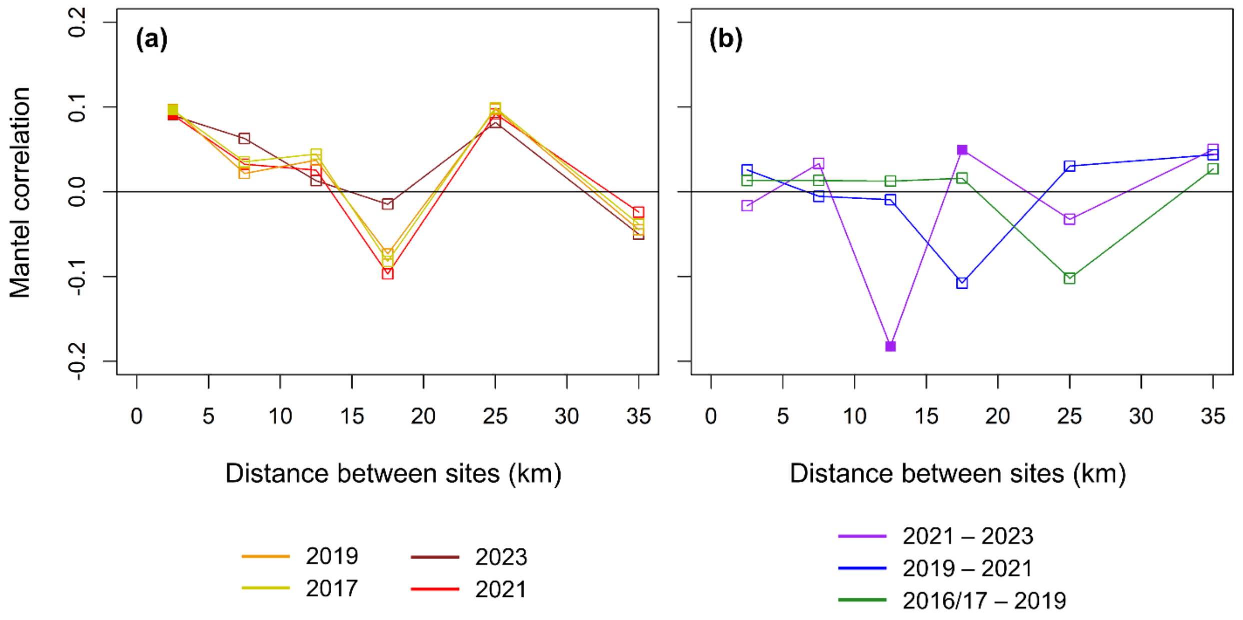

Figure S9. Results of multivariate Mantel correlogram (computed using mantel.correlog in the vegan package) show patterns of spatial autocorrelation between sites based on their (a) community composition in each year of the timeseries, and (b) rates of change in coral cover and rugosity (1cm scale). Multivariate data for (a) consists of percent cover of benthic taxa, structural complexity, and landscape heterogeneity (the same 19 variables as used in the PERMANOVA; Fig. 3). Multivariate data for (b) consists of rates of coral cover and rugosity change (2 variables). We calculated the distance between all sites and dissimilarity between sites, organized site dissimilarities as a function of site distance using 5km bins (0 to 5km, 5 to 10km, etc.), and performed a Mantel correlation for each bin. Significant Mantel correlations are denoted by filled points. (a) For each year of our timeseries, we found a significant positive correlation in community composition for sites within 5km of each other, but no significant evidence of spatial autocorrelation at any other scale. (b) In terms of change in coral cover and rugosity, we found no evidence for spatial autocorrelation between sites at any scale during the first two timesteps (2016/17 – 2019 and 2019 – 2021). In the final timestep (2021 – 2023), we found a significant negative correlation for sites separated by 10 to 15km, and a significant positive correlation for sites separated by 15 to 20km.

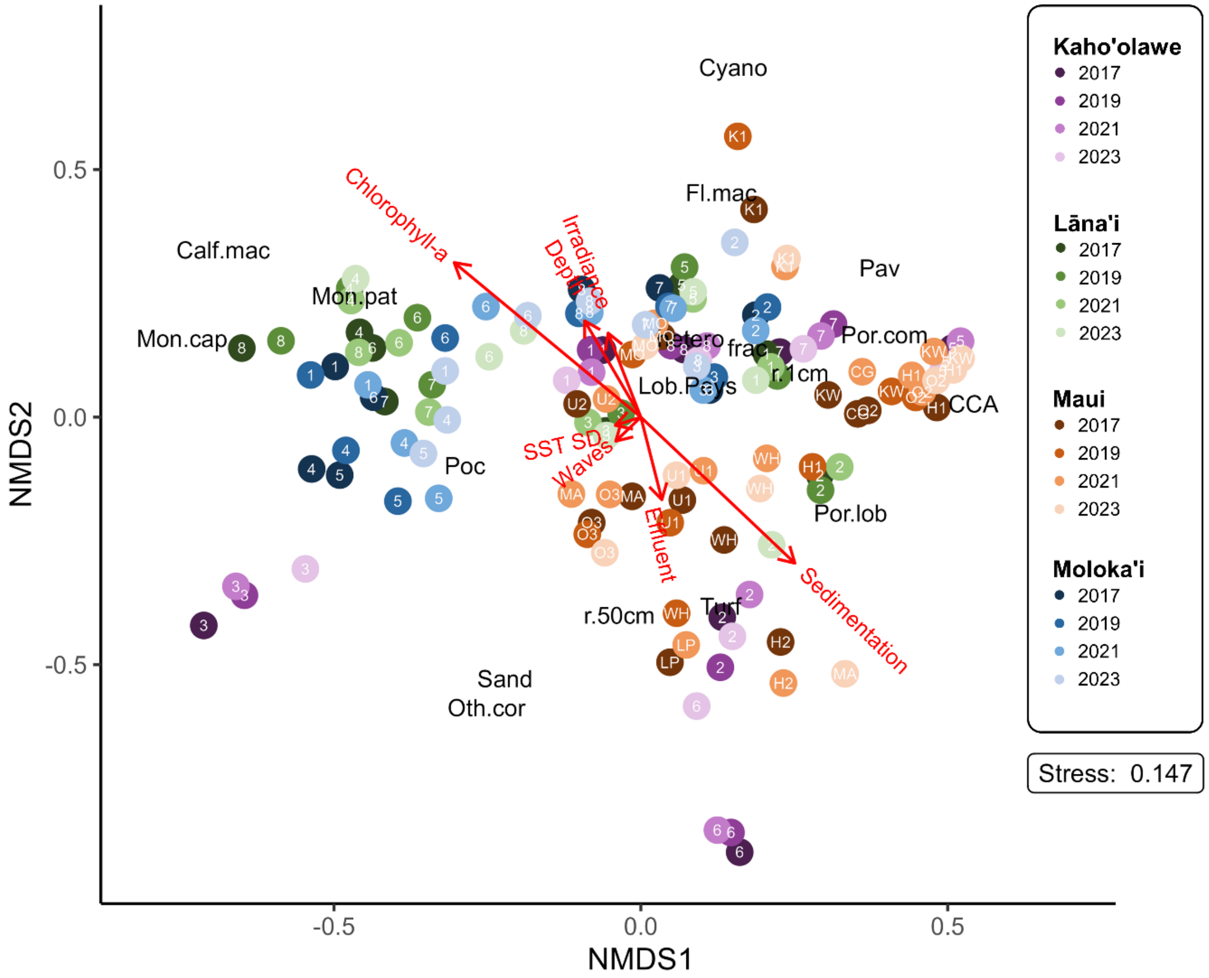

Figure S10. Non-metric Multidimensional Scaling (NMDS) ordination of benthic community composition for all sites in all years. Island is symbolized by point color, with lighter shades representing more recent surveys. The label for each point denotes the site name using the same abbreviations as Fig. 4a and Fig. S1. Red loadings denote environmental variables used in the PERMANOVA, while black text denotes benthic variables used in the ordination. Benthic variables include percent cover of various benthic taxa, structural complexity (r.1cm = rugosity at 1cm resolution; r.50cm = rugosity at 50cm resolution; frac = fractal dimension), and landscape heterogeneity (hetero).

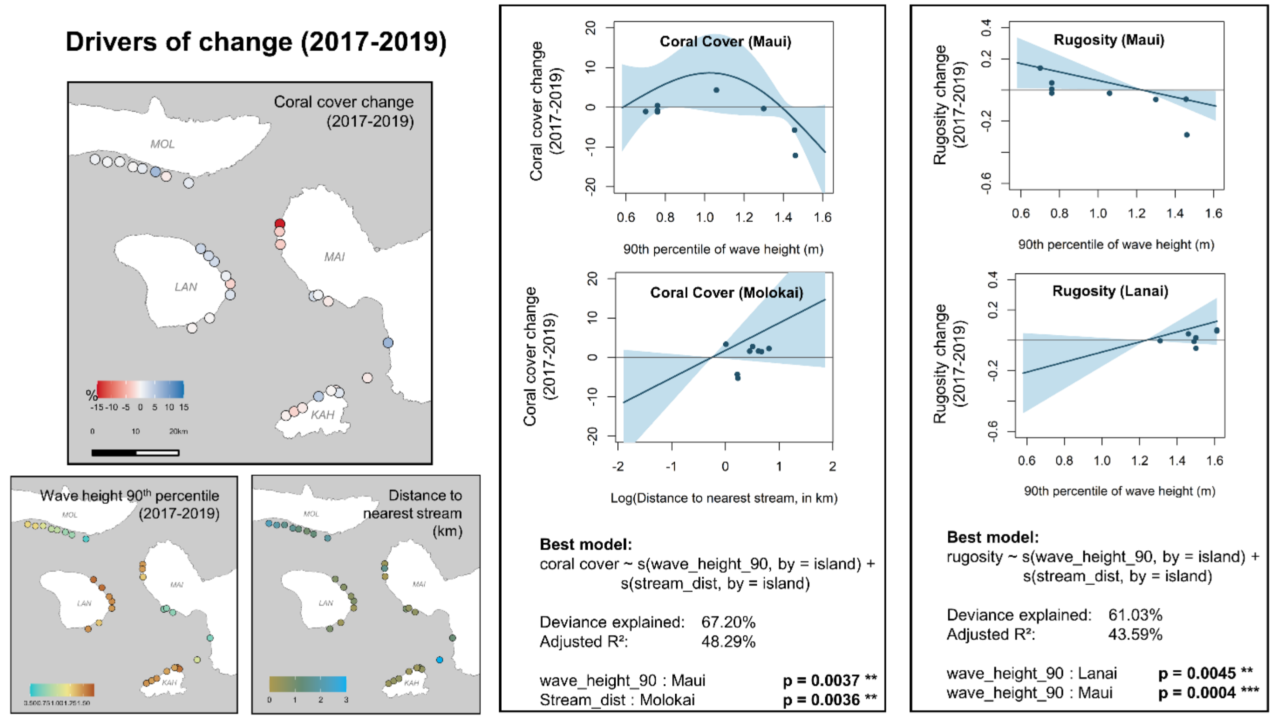

Figure S11. Results of the best generalized additive models (GAM) for timestep 1 (2016/17 – 2019). According to these models, reefs on Maui exposed to moderate wave energy were more likely to see coral cover increase, whereas reefs on Moloka'i located further from streams were more likely to see coral cover increase. Increasing wave exposure was associated with negative rugosity change on Maui but positive rugosity change on Lānaʻi. A map of absolute change in coral cover during timestep 1 is shown, as well as a map of the best environmental predictors in timestep 1. The formulas for the best models (one for coral cover, one for rugosity), deviance explained, adjusted R^2, and p values (significant terms only) are also shown. The relationship between the response variable and significant model terms are illustrated with partial effects plots (shading denotes 95% confidence estimate). See Supplemental Section 3 for full details of GAM parameters and validation.

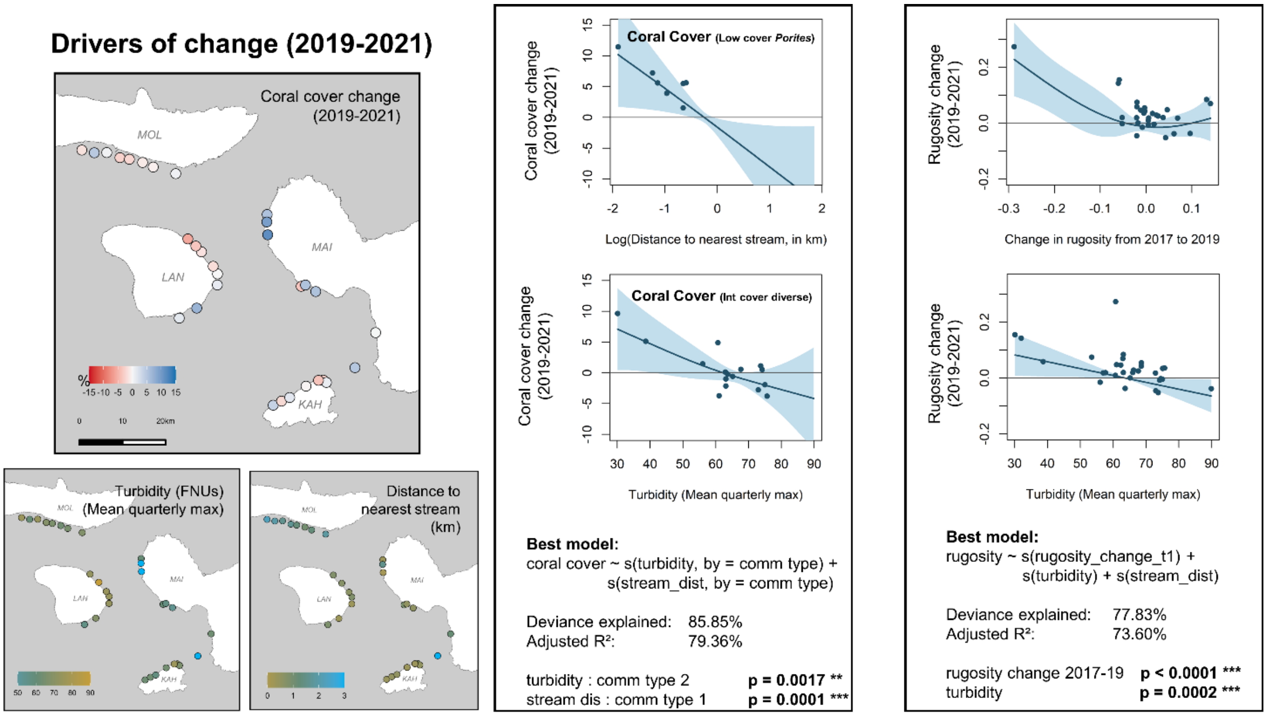

Figure S12. Results of the best generalized additive models (GAM) for timestep 2 (2019 – 2021). According to these models, low cover *Porites* reefs close to streams were more likely to see coral cover increase, while diverse intermediate cover reefs exposed to severe turbidity were more likely to see coral cover decline. Reefs that experienced declines in rugosity from 2017 to 2019 and reefs with less exposure to severe turbidity were more likely to see an increase in rugosity from 2019 to 2021. A map of absolute change in coral cover during timestep 2 is shown, as well as a map of the best environmental predictors in timestep 2. The formulas for the best models (one for coral cover, one for rugosity), deviance explained, adjusted R^2, and p values (significant terms only) are also shown. The relationship between the response variable and significant model terms are illustrated with partial effects plots (shading denotes 95% confidence estimate). See Supplemental Section 3 for full details of GAM parameters and validation.

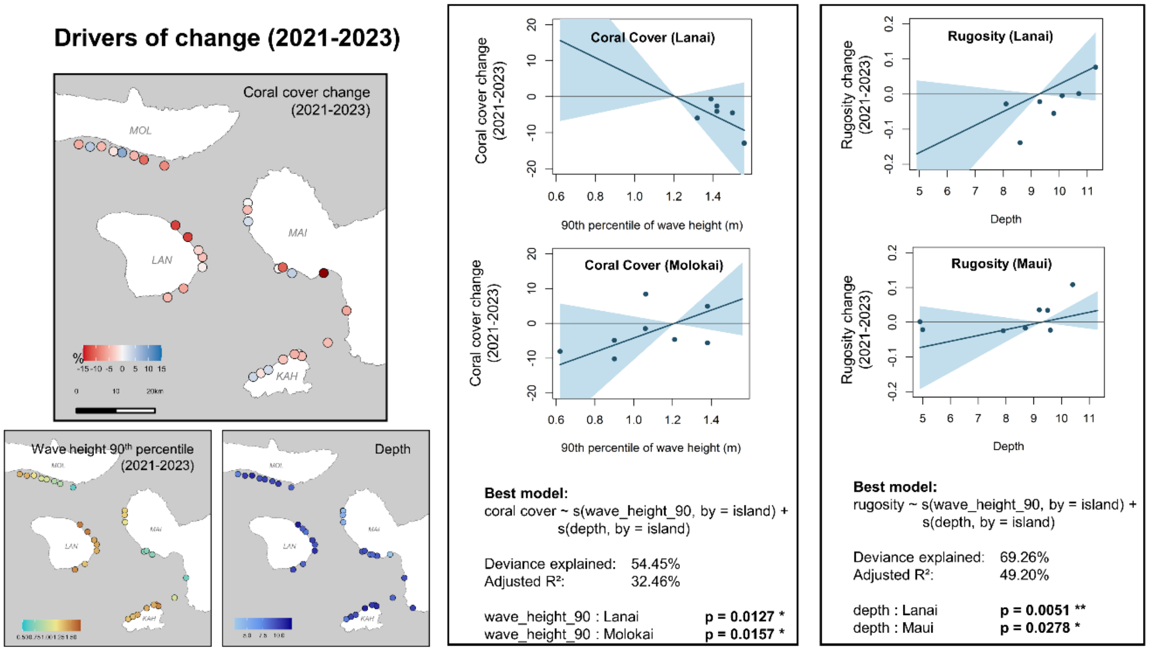

Figure S13. Results of the best generalized additive models (GAM) for timestep 3 (2021 – 2023). According to these models, reefs on Lāna'i exposed to higher wave energy were more likely to see declines in coral cover, whereas reefs on Moloka'i exposed to higher wave energy were more likely to see an increase in coral cover. Rugosity was more likely to increase on deeper reefs on both Maui and Lāna'i. A map of absolute change in coral cover during timestep 3 is shown, as well as a map of the best environmental predictors in timestep 3. The formulas for the best models (one for coral cover, one for rugosity), deviance explained, adjusted R^2, and p values (significant terms only) are also shown. The relationship between the response variable and significant model terms are illustrated with partial effects plots (shading denotes 95% confidence estimate). See Supplemental Section 3 for full details of GAM parameters and validation.

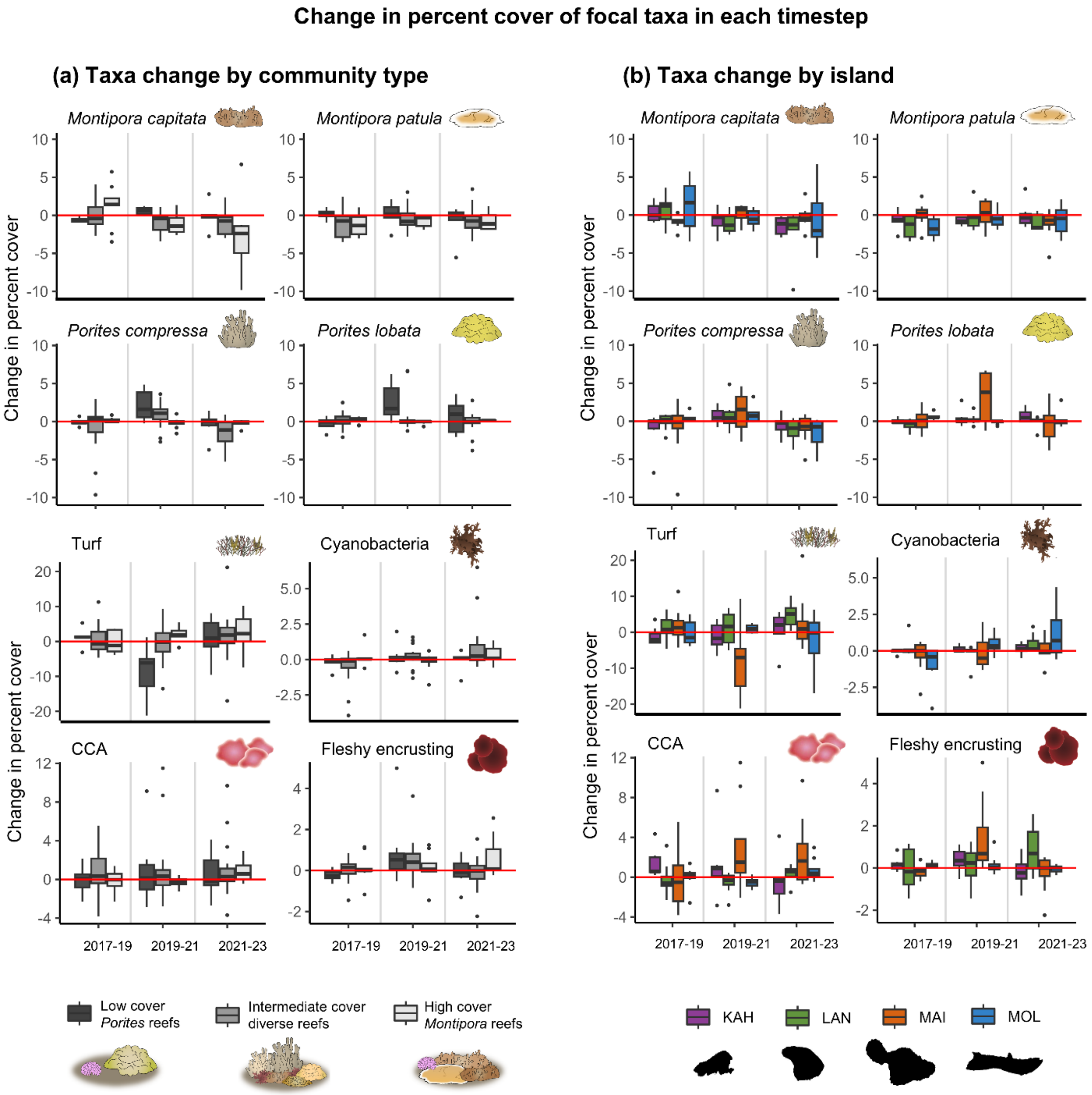

Figure S14. Absolute change in percent cover of key coral taxa and algal functional groups, shown in each timestep by (a) community type and (b) island. Red horizontal line denotes no change during a timestep.

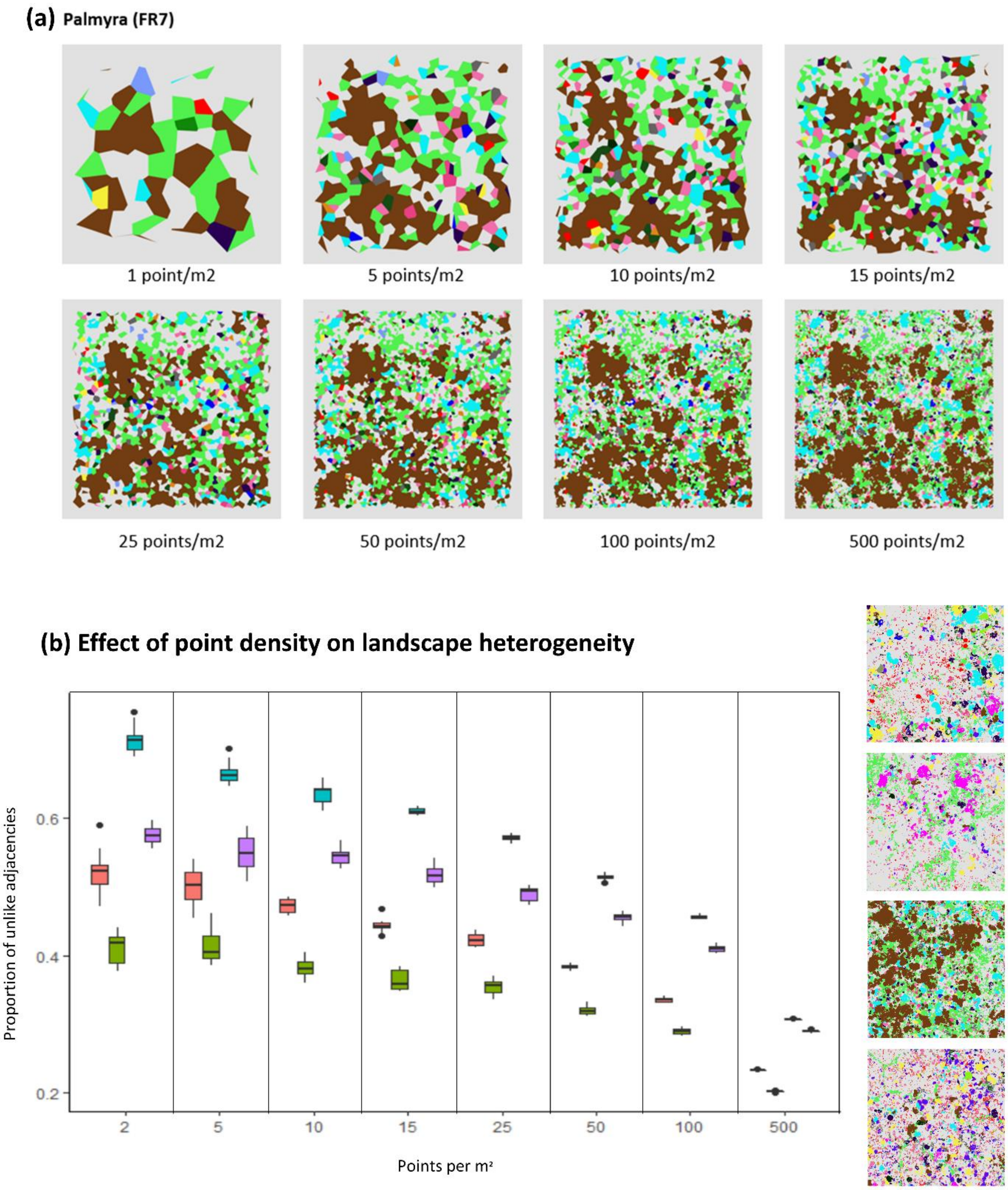

Figure S15. (a) The effect of point density on Voronoi tessellation resolution. A fully traced 10 x 10 m reef plot from Palmyra Atoll (site FR7) is visualized via Voronoi tessellation using eight different point densities. For reference, we used a density of 25 points/m^2 to assess percent cover for sites in Maui Nui. (b) The effect of point density on landscape heterogeneity (represented here as the proportion of unlike adjacencies) is shown for four sites in Palmyra Atoll. We used stratified random sampling to "virtually" assess percent cover 10 times for each fully traced 10 x 10 m reef plot.

Table S1. Results from the repeated-measures PERMANOVA, which we used to explain variance in multivariate community data. The F-statistic, R^2, and p value for eight predictors are shown. For each environmental variable, we compare the range of values at our study sites with the range of values for nearshore environments in the Main Hawaiian Islands, as modeled by the Ocean Tipping Points project (Wedding et al., 2018).

Predictor	*Data descriptor*	*Units*	*Range (our sites)*	*Range (Hawai'i)*	*F statistic*	R^2	*p value*
Island	Fixed factor (4 islands)	--	--	--	16.988	0.227	**< 0.001 *****
Wave power	Max monthly mean (1979-2013)	kW/m	0.8 – 6.4	0 – 140	1.836	0.025	0.273
Depth	Depth at site GPS point	m	3.4 – 11.3	--	2.938	0.039	0.109
SST	Standard deviation (2000-2012)	°C	0.86 – 0.97	0.79 – 1.16	-0.316	-0.004	0.999
Chlorophyll-a	Long-term mean (2002-2013)	mg/m^3	0.06 – 0.12	0.05 – 0.14	1.81	0.024	0.287
Total Effluent	Modeled data	kg/day	0 – 16,085	0 – 118,540	1.142	0.015	0.455
Sediment	Modeled data	tons/ha/yr	0 – 55.7	0 – 516	3.226	0.043	0.088
Irradiance	Long-term mean (2002-2013)	$mol\ m^{-2}\ d^{-1}$	37.4 – 46.2	32.4 – 46.2	6.152	0.082	**0.012 ***

Table S2. Results from mixed-effects linear modelling, which we used to test if community type or island was a better predictor of coral cover change and fine-scale rugosity change between surveys. Community type was a fixed factor with three levels (low cover *Porites* reefs, diverse intermediate cover reefs, and high cover *Montipora* reefs), while island was a fixed factor with four levels (Kaho'olawe, Lāna'i, Maui, and Moloka'i).

Model formula	**AIC**	**p (intercept)**	**p (main effect)**	**p (interaction)**
coral_change ~ community + community : timestep + (1|site)	-10.06	0.988	0.584	**< 0.001** ***
coral_change ~ island + island : timestep + (1|site)	7.07	0.357	0.637	**0.003** **
rugo_change ~ community + community : timestep + (1|site)	-695.41	**0.020** *	0.093	**0.005** **
rugo_change ~ island + island : timestep + (1|site)	-686.90	0.836	0.389	**< 0.001** ***

Table S3. Combinations of explanatory variables used for Generalized Additive Model (GAM) multiple regression analysis. Each combination of variables corresponded to a particular hypothesis explaining change in coral cover or change in fine-scale rugosity in each timestep. Some hypotheses were further combined (i.e., wave event and sedimentation hypothesis combined to form major storm hypothesis), and not all combinations of hypotheses are shown here. Timestep abbreviations are as follows: T1 = 2016/17 – 2019, T2 = 2019 – 2021, and T3 = 2021 – 2023. Figs. S7 – S9 show the results of the best GAM for each timestep (p values, AIC, deviance explained, and adjusted R2 values). Parameters for the best fit models are available in Supplemental Section 3.

**Hypothesis**	**Explanatory variable**	Data source	**Timesteps**
Local-scale environmental and/or human impacts	Island	NA	T1, T2, T3
Benthic community composition	Community type	This study	T1, T2, T3
Succession or recovery dynamics	Change in coral cover or rugosity in previous timestep	This study	T2, T3
Thermal stress	DHW (max, °C/weeks)	(NOAA Coral Reef Watch, 2020)	2015 Max DHW for T1,<br>2019 Max DHW for T2 and T3
Wave event	Wave height (90th percentile, m)<br>Depth (m)	(Cheung, 2021)	T1, T2, T3
Sedimentation	Turbidity (mean of quarterly max, FNU)<br>Distance to nearest stream (m)	(Dogliotti et al. 2015; Hawai'i Statewide GIS Program 2016; Li et al. 2022)	T1 (stream distance only)<br>T2, T3 (turbidity and stream distance)
Major storm (wave event + sedimentation)	Wave height, depth, turbidity, distance to nearest stream	See above	T1, T2, T3

Table S4. Results from analysis of similarities (ANOSIM) of multivariate benthic community data by island and by community type (obtained from hierarchical clustering using the complete linkages method). Abbreviations are as follows: Btwn = between, KAH = Kaho'olawe, LAN = Lāna'i, MAI = Maui, MOL = Moloka'i, C1 = community type 1 (low coral cover *Porites* dominated reefs), C2 = community type 2 (intermediate cover diverse reefs), C3 = community type 3 (high coral cover *Montipora* dominated reefs).

<table>
<tr><th></th><th>By island</th><th>By community type</th></tr>
<tr><td>ANOSIM statistic (R)</td><td>0.2415</td><td>0.6932</td></tr>
<tr><td>p value</td><td>< 0.001 ***</td><td>< 0.001 ***</td></tr>
<tr><td>Upper quantiles of permutations (null model)</td>
<td><table><tr><th>90%</th><th>95%</th><th>97.5%</th><th>99%</th></tr><tr><td>0.0202</td><td>0.0279</td><td>0.0342</td><td>0.0420</td></tr></table></td>
<td><table><tr><th>90%</th><th>95%</th><th>97.5%</th><th>99%</th></tr><tr><td>0.0415</td><td>0.0544</td><td>0.0673</td><td>0.0809</td></tr></table></td></tr>
<tr><td>Dissimilarity ranks between and within groups</td>
<td><table><tr><th></th><th>0%</th><th>25%</th><th>50%</th><th>75%</th><th>100%</th><th>N</th></tr>
<tr><td>Btwn.</td><td>96</td><td>2563</td><td>4738</td><td>7005</td><td>8911</td><td>6669</td></tr>
<tr><td>KAH</td><td>1</td><td>1644</td><td>5528</td><td>7492</td><td>8848</td><td>378</td></tr>
<tr><td>LAN</td><td>4</td><td>1304</td><td>3532</td><td>5638</td><td>8680</td><td>465</td></tr>
<tr><td>MAI</td><td>9</td><td>1691</td><td>3591</td><td>5315</td><td>8249</td><td>903</td></tr>
<tr><td>MOL</td><td>2</td><td>1055</td><td>2553</td><td>5121</td><td>8034</td><td>496</td></tr></table></td>
<td><table><tr><th></th><th>0%</th><th>25%</th><th>50%</th><th>75%</th><th>100%</th><th>N</th></tr>
<tr><td>Btwn.</td><td>238</td><td>4083</td><td>5997</td><td>7549</td><td>8911</td><td>5340</td></tr>
<tr><td>C1</td><td>7</td><td>1162</td><td>2508</td><td>3838</td><td>7703</td><td>435</td></tr>
<tr><td>C2</td><td>1</td><td>955</td><td>2159</td><td>3896</td><td>8201</td><td>2701</td></tr>
<tr><td>C3</td><td>19</td><td>818</td><td>1981</td><td>4351</td><td>7981</td><td>435</td></tr></table></td></tr>
</table>

References

Bak, R. P. M., & Meesters, E. H. (1998). Coral population structure: The hidden information of colony size-frequency distributions. *Marine Ecology Progress Series*, *162*, 301–306. https://doi.org/10.3354/meps162301

Barott, K. L., Williams, G. J., Vermeij, M. J. A., Harris, J., Smith, J. E., Rohwer, F. L., & Sandin, S. A. (2012). Natural history of coral-algae competition across a gradient of human activity in the Line Islands. *Marine Ecology Progress Series*, *460*, 1–12. https://doi.org/10.3354/meps09874

Cheung, K. F. (2021). *WaveWatch III (WW3) Global Wave Model. July 2016 to July 2023.* Distributed by the Pacific Islands Ocean Observing System (PacIOOS). http://pacioos.org/metadata/ww3_global.html.

Connell, J. H. (1978). Diversity in tropical rain forests and coral reefs. *Science*, *199*, 1302–1309. https://doi.org/10.1126/science.199.4335.1302

Cushman, S. A., McGarigal, K., & Neel, M. C. (2008). Parsimony in landscape metrics: Strength, universality, and consistency. *Ecological Indicators*, *8*(5), 691–703. https://doi.org/10.1016/j.ecolind.2007.12.002

Dietzel, A., Connolly, S. R., Hughes, T. P., & Bode, M. (2021). The spatial footprint and patchiness of large-scale disturbances on coral reefs. *Global Change Biology*, *27*(19), 4825–4838. https://doi.org/10.1111/gcb.15805

Dogliotti, A. I., Ruddick, K. G., Nechad, B., Doxaran, D., & Knaeps, E. (2015). A single algorithm to retrieve turbidity from remotely-sensed data in all coastal and estuarine waters. *Remote Sensing of Environment*, *156*, 157–168. https://doi.org/10.1016/j.rse.2014.09.020

Dollar, S. J. (1982). Wave Stress and Coral Community Structure in Hawaii. *Coral Reefs*, *I*, 71–81. https://doi.org/10.1007/BF00301688

Edwards, C. B., Eynaud, Y., Williams, G. J., Pedersen, N. E., Zgliczynski, B. J., Gleason, A. C. R. R., Smith, J. E., & Sandin, S. A. (2017). Large-area imaging reveals biologically driven non-random spatial patterns of corals at a remote reef. *Coral Reefs*, *36*(4), 1291–1305. https://doi.org/10.1007/s00338-017-1624-3

Fox, M. D., Carter, A. L., Edwards, C. B., Takeshita, Y., Johnson, M. D., Petrovic, V., Amir, C. G., Sala, E., Sandin, S. A., & Smith, J. E. (2019). Limited coral mortality following acute thermal stress and widespread bleaching on Palmyra Atoll, central Pacific. *Coral Reefs*, *38*(4), 701–712. https://doi.org/10.1007/s00338-019-01796-7

George, E. E., Mullinix, J. A., Meng, F., Bailey, B. A., Edwards, C., Felts, B., Haas, A. F., Hartmann, A. C., Mueller, B., Roach, T. N. F., Salamon, P., & Silveira, C. (2021). Space-filling and benthic competition on coral reefs. *PeerJ*, *9*, 1–25. https://doi.org/10.7717/peerj.11213

Grigg, R. W. (1983). Community structure, succession and development of coral reefs in Hawaii. *Marine Ecology Progress Series*, *11*, 1–14. https://doi.org/10.3354/meps011001

Haire, S. L., & McGarigal, K. (2009). Changes in fire severity across gradients of climate, fire size, and topography: A landscape ecological perspective. *Fire Ecology*, *5*(2), 86–103. https://doi.org/10.4996/fireecology.0502086

Hawaii Statewide GIS Program. (2016). *Streams - Hawaii*. Distributed by the Pacific Islands Ocean Observing System (PacIOOS). http://pacioos.org/metadata/hi_hcgg_all_darstreams.html

Kindlmann, P., & Burel, F. (2008). Connectivity measures: A review. *Landscape Ecology*, *23*(8), 879–890. https://doi.org/10.1007/s10980-008-9245-4

Krummel, J. R., Gardner, R. H., Sugihara, G., O'Neill, R. V, & Coleman, P. R. (1987). Landscape patterns in a disturbed environment. *Oikos*, *48*(3), 321–324. https://doi.org/10.2307/3565520

Li, J., Carlson, R. R., Knapp, D. E., & Asner, G. P. (2022). Shallow coastal water turbidity monitoring using Planet Dove satellites. *Remote Sensing in Ecology and Conservation*, *8*(4), 521–535. https://doi.org/10.1002/rse2.259

McCarthy, O. S., Smith, J. E., Petrovic, V., & Sandin, S. A. (2022). Identifying the drivers of structural complexity on Hawaiian coral reefs. *Marine Ecology Progress Series*, *702*, 71–86. https://doi.org/10.3354/meps14205

McGarigal, K., & Cushman, S. A. (2002). Comparative evaluation of experimental approaches to the study of habitat fragmentation effects. *Ecological Applications*, *12*(2), 335–345. https://doi.org/10.1890/1051-0761(2002)012[0335:CEOEAT]2.0.CO;2

Nash, K. L., Graham, N. A. J., Wilson, S. K., & Bellwood, D. R. (2013). Cross-scale habitat structure drives fish body size distributions on coral reefs. *Ecosystems*, *16*(3), 478–490. https://doi.org/10.1007/s10021-012-9625-0

NOAA Coral Reef Watch. (2020). *NOAA Coral Reef Watch Operational Daily Near-Real-Time Global 5-km Satellite Coral Bleaching Monitoring Products. Version 3.1. July 2014 to July 2023.* Distributed by the Pacific Islands Ocean Observing System (PacIOOS). http://pacioos.org/metadata/dhw_5km.html

Pittman, S., Kneib, R., & Simenstad, C. (2011). Practicing coastal seascape ecology. *Marine Ecology Progress Series*, *427*. https://doi.org/10.3354/meps09139

Price, D. M., Lim, A., Callaway, A., Eichhorn, M. P., Wheeler, A. J., Lo Iacono, C., & Huvenne, V. A. I. (2021). Fine-Scale Heterogeneity of a Cold-Water Coral Reef and Its Influence on the Distribution of Associated Taxa. *Frontiers in Marine Science*, *8*. https://doi.org/10.3389/fmars.2021.556313

Rodriguez, C., Amir, C., Gray, A., Asbury, M., Suka, R., Lamirand, M., Couch, C., Oliver, T., Rodriguez, C., Amir, C., Gray, A., & Asbury, M. (2021). *Measuring Coral Vital Rates Using Photogrammetry at Fixed Sites : Standard Operating Procedures and Error Estimates Measuring Coral Vital Rates Using Structure-from-Motion Photogrammetry at Fixed Sites : Standard Operating Procedures and Error Estimates*. https://doi.org/10.25923/a9se-k649

Šímová, P., & Gdulová, K. (2012). Landscape indices behavior: A review of scale effects. *Applied Geography*, *34*, 385–394. https://doi.org/10.1016/j.apgeog.2012.01.003

Turner, M. G., Baker, W. L., Peterson, C. J., & Peet, R. K. (1998). Factors Influencing Succession: Lessons from Large, Infrequent Natural Disturbances. *Ecosystems*, *1*, 511–523. https://doi.org/10.1007/s100219900047

Turner, M. G., O'Neill, R. V, Gardner, R. H., & Milne, B. T. (1989). Effects of changing spatial scale on the analysis of landscape pattern. *Landscape Ecology*, *3*, 153–162. https://doi.org/10.1007/BF00131534

Wedding, L., Lepczyk, C., Pittman, S., Friedlander, A., & Jorgensen, S. (2011). Quantifying seascape structure: extending terrestrial spatial pattern metrics to the marine realm. *Marine Ecology Progress Series*, *427*. https://doi.org/10.3354/meps09119

Wedding, L. M., Lecky, J., Gove, J. M., Walecka, H. R., Donovan, M. K., Williams, G. J., Jouffray, J. B.,

Crowder, L. B., Erickson, A., Falinski, K., Friedlander, A. M., Kappel, C. V., Kittinger, J. N., McCoy, K., Norström, A., Nyström, M., Oleson, K. L. L., Stamoulis, K. A., White, C., & Selkoe, K. A. (2018). Advancing the integration of spatial data to map human and natural drivers on coral reefs. *PLoS ONE*, *13*(3). https://doi.org/10.1371/journal.pone.0189792

Williams, G. J., Smith, J. E., Conklin, E. J., Gove, J. M., Sala, E., & Sandin, S. A. (2013). Benthic communities at two remote pacific coral reefs: Effects of reef habitat, depth, and wave energy gradients on spatial patterns. *PeerJ*, *2013*(1), 1–26. https://doi.org/10.7717/peerj.81

Wood, S. N. (2011). Fast stable restricted maximum likelihood and marginal likelihood estimation of semiparametric generalized linear models. *Journal of the Royal Statistical Society (B)*, *73*(1), 3–36. https://doi.org/10.1111/j.1467-9868.2010.00749.x
